# Oxidative stress, aging, metabolism, SIRT1, and the gut microbiota: the neurocardiac basis of cognitive loss

**DOI:** 10.4103/mgr.MEDGASRES-D-26-00073

**Published:** 2026-08-03

**Authors:** Kenneth Maiese

**Affiliations:** Cellular and Molecular Signaling, New York, NY, USA

**Keywords:** Alzheimer’s disease, APOE, cardiac disease, cell senescence, dementia, diabetes mellitus, GLP-1, gut microbiota, mechanistic target of rapamycin (mTOR), mitochondria, multiple sclerosis, oxidative stress, programmed cell death, silent mating type information regulation 2 homolog 1 (SIRT1)

## Abstract

Cardiovascular disease and cognitive loss have a neurocardiac basis. Poor vascular perfusion can impair cognitive function in both Alzheimer’s disease and multiple sclerosis. However, a treatment gap exists because current approaches do not adequately address the shared underlying cellular mechanisms responsible for cognitive dysfunction in these conditions. Current treatments for cognitive impairment in diseases such as cardiovascular disease, Alzheimer’s disease, multiple sclerosis, and diabetes often fail to fully address the shared underlying cellular mechanisms. Consequently, the prevailing precision treatment strategy, which focuses on managing symptoms and preventing disease progression, is insufficient. This highlights the urgent need for innovative approaches capable of targeting these common cellular pathways across these diverse conditions. Novel investigations into oxidative stress, cellular senescence, programmed cell death with apoptosis, ferroptosis, pyroptosis, and autophagy, cellular metabolism with apolipoprotein E and glucagon-like peptide-1 receptor agonism, silent mating type information regulation 2 homolog 1 (*Saccharomyces cerevisiae*), mitochondrial dynamics, and the gut microbiome offer the potential to address the risk factors and clinical treatments for cardiovascular disease and cognitive loss. These pathways are exquisitely dependent upon one another and require in-depth knowledge of the modulatory cellular mechanisms for effective translation to clinical care.

## Introduction

With recent improvements in healthcare across the globe, which include improved public health policies, greater access to acute and chronic healthcare, and early disease detection and treatment,^1^,^2^,^3^,^4^,^5^,^6^,^7^,^8^,^9^,^10^,^11^,^12^,^13^,^14^ lifespan has increased worldwide to the extent that by the year 2030, almost 20% of individuals will be over the age of 60 years, and by the year 2050, more than 425 million individuals will be over the age of 80 years.^7^,^15^,^16^,^17^,^18^ As a result of the global rise in aging and lifespan, a parallel increase in non-communicable diseases (NCDs) has occurred, with 40 million people annually succumbing to NCDs as a result of leading causes of death from cardiovascular disease^19^,^20^,^21^ and Alzheimer’s disease (AD).^22^,^23^,^24^,^25^,^26^ Interestingly, there is a neurocardiac relationship between cardiovascular disease and cognitive loss in patients with AD, as well as other memory-associated disorders, such as multiple sclerosis (MS). The neurocardiac relationship is the close network of system and cellular pathways that connect and influence the cardiovascular system and the nervous system. Disorders of the cardiovascular system that include angina, congestive heart failure, myocardial infarction, microvascular injury, endothelial cell injury, platelet dysfunction, and susceptibility to viral infections with severe acute respiratory syndrome coronavirus 2 (SARS-CoV-2) and coronavirus disease 2019 (COVID-19) have been associated with the development of memory loss, intellectual disability, and behavioral health illnesses.^27^,^28^,^29^,^30^,^31^,^32^,^33^,^34^,^35^ In AD, poor perfusion from an impaired cardiac system can cause hypoperfusion to the brain, but the underlying cellular pathways need to be investigated to identify pathological targets and treatment regimens that ultimately lead to AD, which can involve amyloid and tau deposition, loss of metabolic function, and inflammatory cell microglial activity. In a similar respect to MS, cardiac rhythm irregularities and loss of cerebral perfusion can affect cognitive and motor function, but the underlying cellular mechanisms require future clinical care given that these mechanisms are similar to those of AD and lead to amyloid and tau deposition in the brain to foster cognitive and motor loss and impair oligodendrocyte maturation to lead to demyelination.^36^,^37^,^38^ These cardiovascular disease and cognitive loss disorders are associated with cellular metabolic dysfunction with diabetes mellitus (DM), which can lead to heart failure, decreased cerebral blood flow, insulin resistance, glucose intolerance, perivascular pathway impairment, blood–brain barrier failure, and inflammation.^39^,^40^,^41^,^42^,^43^,^44^,^45^,^46^

Several new clinical therapies and care pathways for cardiovascular disease, AD, and DM are emerging. This neurocardiac and metabolic axis encompasses the functional network that connects the cardiovascular system, the nervous system, and metabolic homeostasis, which ultimately can impact cognitive function. Cardiovascular disease risk can be reduced through the management of blood pressure elevation, serum cholesterol reduction, improved nutrition, regular exercise, minimal use or elimination of tobacco and alcohol consumption, a reduction in exposure to environmental toxins, and appropriate weight control. Additional care pathways for cardiac disease, such as those for endothelial dysfunction and atherosclerosis, offer early disease detection.^47^,^48^ With respect to patients with cognitive loss with conditions such as AD and MS, treatments rely upon lifestyle changes such as cognitive exercises, physical exercise, cholinesterase inhibitors, monitoring with wearable biosensors, new immunotherapies designed to clear brain amyloid-β (Aβ) deposition, and disease-modifying therapies for MS to reduce relapses in patients who are relapsing–remitting of the disease. DM care includes nutritional care, weight management, regular physical exercise, and pharmaceutical agents to control hyperglycemia and hypoglycemia. However, these current therapies for cardiovascular disease, AD, and DM are targeted for symptomatic relief and cannot prevent the eventual progression of the underlying disease process. Furthermore, these therapies are not without potential complications and can involve brain microhemorrhages, brain volume loss, and the comorbidity of off-target disabilities that result in cardiac and neuronal cell injury and organ atrophy.^34^,^49^,^50^,^51^,^52^,^53^ With these challenges, innovative studies that consider oxidative stress, cellular senescence, programmed cell death with apoptosis, ferroptosis, pyroptosis, and autophagy, cellular metabolism with apolipoprotein E (APOE) and glucagon-like peptide-1 (GLP-1) receptor agonism, silent mating type information regulation 2 homolog 1 (*Saccharomyces cerevisiae*) (SIRT1), mitochondrial dynamics, and the gut microbiome provide exciting prospects to address the shared underlying mechanisms for cardiovascular disease and cognitive loss that form the critical neurocardiac foundation for the development of vital treatment strategies for these disorders. This review aimed to identify the shared cellular pathways linking cardiovascular disease, cognitive loss, and diabetes mellitus, thereby bridging the treatment gap beyond symptomatic care toward mechanism-based strategies.

## Data Sources

Data sources were selected on the basis of a literature search using the PubMed, Scopus, Web of Science, and ScienceDirect databases from January 2021 through March 2026. The search terms included “cardiovascular disease,” “dementia,” “neurodegenerative,” “Alzheimer’s disease,” “cardiac,” “gut microbiota,” “aging,” “metabolism,” “diabetes mellitus,” “programmed cell death,” “oxidative stress,” “sirtuin,” and “mitochondria” with Boolean operators (AND, OR) to focus the search strategy. Peer-reviewed original research and review papers, including the citations in the review papers, were included, while non-peer reviewed works, duplicate studies, unrelated studies, and abstracts or meeting presentations with incomplete information were excluded.

## Lifespan Extension, Aging, and Non-communicable Diseases

Throughout the global community, lifespan has been increasing in both developed and developing countries.^54^,^55^,^56^ One factor that has contributed to an increased lifespan is the decrease in overdoses of drug use and the decline in deaths from viral infections with SARS-CoV-2 and COVID-19.^33^,^57^,^58^,^59^ Other factors include the availability of educational programs to promote healthy nutrition and cardiovascular training, greater access to local and tertiary healthcare centers, improved public healthcare policies that target disease prevention, a reduction in environmental toxins, and enhanced sanitation, and the development of sophisticated diagnostics for early disease detection and nutritional analysis.^1^,^2^,^3^,^4^,^5^,^6^,^7^,^8^ In developed nations, the number of individuals who are older than 65 years has increased by more than 100% over the past 50 years. Furthermore, in developing nations, the population of individuals over the age of 65 years is expected to increase by 10%. By the year 2030, at least 17% of individuals worldwide will be over the age of 60 years, and by the year 2050, approximately 80% of the elderly population will reside in low- and middle-income countries as the aging of the population rapidly increases.^49^,^56^,^60^ During the next 20 years, the number of individuals aged over 60 years will increase from 1.4 billion people to 2.1 billion people, with the expected lifespan reaching 80 years of age for most individuals. At this point, the number of individuals over the age of 80 years is expected to exceed 425 million people.^7^,^15^,^16^,^17^,^18^

With the global increase in lifespan and aging, there has been a corresponding rise in NCDs. Although NCDs are chronic disorders that are linked to aging, nearly 20 million deaths related to NCDs can occur before the age of 70 years. Approximately 75% of deaths related to NCDs occur in low- and middle-income countries, and over 40 million people die annually from NCDs. More than 30% of individuals less than 60 years of age will suffer from NCDs in low- and middle-income countries, while only 10% of individuals in high-income countries will succumb to NCDs.^55^,^61^ Poor nutrition, a lack of physical activity, tobacco and alcohol use, air pollution exposure, hypertension, obesity, metabolic disorders such as DM, and elevated serum lipid levels with high cholesterol levels all contribute to the risk of developing NCDs. NCDs include cardiovascular diseases that involve myocardial infarction and stroke; dementia, which includes AD; cancer; musculoskeletal disease, which involves osteoarthritis; trauma; reproductive disorders; pulmonary disease; metabolic disorders with DM; and mental health disorders, which can include suicide and renal disease.^61^,^62^,^63^,^64^,^65^,^66^,^67^,^68^,^69^ As a result of increased aging of the population with increasing lifespan, the neurocardiac axis is affected by increased cardiac failure, vascular disease, valvular dysfunction, increased arrhythmias, atherosclerosis, hypertension, and elevated oxidative stress.^20^,^21^,^39^,^70^,^71^,^72^,^73^,^74^ Similarly, the nervous system can undergo atrophy of the brain, degeneration of myelin, increased levels of neuronal intracellular toxins, impairments in motor and sensory function, and cognitive dysfunction.^54^,^72^,^75^,^76^,^77^,^78^,^79^,^80^,^81^,^82^ Loss of metabolic homeostasis is an important component of the aging of the neurocardiac axis and leads to insulin resistance, inflammation, body weight changes, cellular senescence, and mitochondrial dysfunction.^39^,^47^,^66^,^68^,^83^,^84^,^85^,^86^,^87^,^88^,^89^,^90^,^91^,^92^,^93^,^94^,^95^,^96^,^97^

## Cardiovascular Disease, Oxidative Stress, Cell Senescence, and Programmed Cell Death

In regard to NCDs, cardiovascular disorders are the leading cause of death for men and women, with over 20 million deaths annually, approximately one-third of the deaths that occur throughout the world.^9^,^98^,^99^,^100^ Almost 90% of these deaths are the result of myocardial infarction and stroke, with 75% of these deaths occurring in low- and middle-income countries.^101^ With respect to individuals under the age of 70 years, approximately 40% of deaths from NCDs are a result of cardiovascular disease, and compared with women, men can succumb to cardiovascular disease earlier, which occurs at the age of 35 years.^102^ In the US alone, it is reported that one person succumbs to cardiovascular disease every 33 seconds. Similar to the risk factors for NCDs, cardiovascular disease risk factors include a lack of nutritional care, a lack of physical activity, tobacco and alcohol use, air pollution exposure, hypertension, obesity, metabolic disease, such as DM, increased serum lipids with cholesterol, and infection.^43^,^103^,^104^,^105^,^106^,^107^,^108^,^109^,^110^ Financial costs for cardiovascular care are a significant challenge for all nations. In the US, direct medical costs for cardiovascular care exceed 420 billion US dollars, with average direct medical costs per patient equal to almost 19,000 US dollars. Projected costs for cardiovascular care by the year 2034 are expected to exceed 1.45 trillion US dollars.^111^,^112^

Oxidative stress and the generation of reactive oxygen species (ROS) are significant components of cardiovascular disease and aging.^113^,^114^,^115^,^116^,^117^,^118^ ROS include free radical species such as nitric oxide, singlet oxygen, hydrogen peroxide, superoxide free radicals, and peroxynitrite.^49^,^66^,^119^,^120^,^121^ At the cellular level, antioxidant systems that involve superoxide dismutase, vitamins C, K, E, B, and D, glutathione peroxidase, and catalase serve to block the detrimental effects of ROS.^11^,^87^,^122^,^123^,^124^,^125^,^126^,^127^,^128^ If cellular antioxidant systems become incapable of reducing oxidative stress during disease onset in the cardiovascular system, then multiple disorders can result, including cardiomyopathy, stem cell loss, atherosclerosis, cardiomyocyte cell loss, programmed cell death, and inflammation.^41^,^93^,^113^,^114^,^115^,^116^,^129^,^130^,^131^,^132^,^133^,^134^,^135^ During cardiovascular events such as myocardial infarction, oxidative stress can lead to cell death, endothelial cell loss, cardiomyocyte death, cellular energy depletion with mitochondrial stress, and programmed cell death through autophagy, ferroptosis, and pyroptosis.^116^,^129^,^131^,^136^,^137^ Notably, pathways associated with oxidative stress that lead to cell death are complex and dependent upon the induction of closely linked cellular mechanisms. For example, oxidative stress injury can be tissue- and sex-specific, reliant upon cellular energy pathways with the coenzyme β-nicotinamide adenine dinucleotide (NAD^+^), linked to inflammatory cell activity that affects immune homeostasis, and requires restorative treatments targeted to maintain redox energy balance.^18^,^138^,^139^,^140^,^141^

With the onset of oxidative stress, processes involving progressive aging occur and initiate cellular senescence.^142^,^143^,^144^,^145^,^146^,^147^ Cellular senescence is involved chronic inflammation, mitochondrial dysfunction, autophagy impairment, cardiomyocyte apoptotic cell death, gut microbiota changes, cellular metabolic disturbances, energy loss, fibrosis, downregulation of gene transcription, and altered lipid metabolism.^56^,^89^,^113^,^133^,^143^,^145^,^148^,^149^,^150^,^151^,^152^,^153^,^154^,^155^,^156^ The onset of cellular senescence is mediated by dysfunction in telomere (TL) processing.^7^,^39^,^142^ TLs are complexes of DNA that control the replication and survival of cells through the protection of genomic DNA.^39^,^112^,^147^,^148^,^157^ TLs exist on chromosome ends and contain more than 2000 repetitions of non-coding double-stranded DNA, are finalized at the ends with guanine-rich single-stranded DNA, and have the sequence “TTAGGG.” As components of the TL group, the protein complexes shelterin, telosome, and CTC1-STN1-TEN1 control TL activity and stability.^158^ With the oversight of the effects of TLs on cell replication, telomerase protein functions to limit the loss of base pairs in TLs and becomes active during cell division, which can result in the loss of 25–200 bp during this process. Telomerase adds tandem repeat RNA templates for the maintenance of TL length. However, as time progresses, TLs lose function, which can involve telomerase impairment, with TLs having less than 500 bp in length, cell senescence progresses in the absence of cellular replication, and the induction of oxidative stress is promoted by energy impairment and mitochondrial dysfunction.^23^,^131^,^137^,^159^,^160^,^161^,^162^,^163^,^164^,^165^,^166^,^167^,^168^

Closely linked to the pathways involved in cellular senescence and oxidative stress is the induction of programmed cell death, which includes apoptosis, ferroptosis, pyroptosis, and autophagy. Apoptosis can lead to cell death through an initial juncture that requires membrane phosphatidylserine (PS) residue externalization. Once this process occurs, inflammatory cells, such as microglia, seek injured cells with PS externalization to remove these cells.^169^,^170^,^171^,^172^,^173^ As the initial presentation of apoptotic cell death, this particular phase of PS externalization is reversible as a potential point of treatment through the blockade of PS externalization and prevention of microglial phagocytosis to rescue injured cells destined for apoptotic cell death.^174^,^175^,^176^,^177^ In the process of cell survival, microglia can play variable roles, such as during AD, to remove Aβ deposits,^178^,^179^,^180^,^181^,^182^ but they can also promote oxidative stress.^138^,^183^,^184^,^185^ Triggering receptor expressed on myeloid cells 2, which can promote microglial cell survival, can also reduce inflammation and oxidative stress in environments that otherwise lead to cell death.^7^,^186^,^187^ Following the juncture with membrane PS externalization, a second stage of apoptotic cell death proceeds that is irreversible with mitochondrial membrane depolarization, release of cytochrome c, activation of caspase pathways, and DNA degradation.^114^,^164^,^188^,^189^,^190^ During oxidative stress, apoptosis can lead to cardiomyocyte death in the ischemic myocardium,^191^ the death of cardiac tissue during diabetic cardiomyopathy,^114^,^117^ the loss of cardiac cells during exposure to environmental toxins,^115^ the absence of cardiac cell regeneration during metabolic dysfunction,^192^ the promotion of cardiac aging during ROS exposure,^167^ and myocardial susceptibility during ischemia–reperfusion injury.

Ferroptosis results in programmed cell death through mechanisms that result in excessive cellular iron storage with the loss of glutathione homeostasis. In the absence of the ability of cells to protect themselves through glutathione-based systems, lipid peroxidation results in cell death.^49^,^193^,^194^,^195^,^196^,^197^ Ischemia–reperfusion injury can lead to cell death through ferroptosis,^198^ and ferroptosis also promotes decreased cell survival during myocardial infarction.^116^ During cardiotoxicity with doxorubicin, ferroptosis results in cardiomyocyte cell death as well.^199^

In regard to pyroptosis, programmed cell death is overseen by the inflammasome, also termed the pyroptosome, which modulates inflammation.^116^,^166^,^200^,^201^ The inflammasome family of nucleotide-binding oligomerization domain and leucine-rich repeat-containing receptors (NLRs) contains NLRP1, NLRP3, NLRP6, and NLRC4 as members and activates inflammasomes. NLRs ultimately activate caspase 1, caspase 4, and caspase 5 through pattern recognition receptors that respond to damage-associated molecular patterns (DAMPs) and through pathogen-associated molecular patterns (PAMPs). Gasdermin proteins, activated by caspase 1, generate membrane pores through their N-terminal domains following cleavage from the C-terminal domains, fostering DAMP molecules such as DNA and adenosine triphosphate (ATP) to pass through the open cell membrane. As a result, activation of the canonical NLRP3 inflammasome occurs, cytokines such as interleukin-1 family members are released, and caspase 4 and caspase 5 activate noncanonical inflammasomes with lipopolysaccharide proteins during infectious processes in gram-negative bacteria.^179^,^186^,^202^,^203^,^204^ Pyroptosis is activated during oxidative stress, which can occur with myocardial infarction and result in cardiac cell death.^116^ In cardiomyopathy with DM, the expression of gasdermin is increased by NLRP3, and the induction of pyroptosis subsequently results in cardiomyocyte injury^200^,^205^ in conjunction with insulin resistance and glucose intolerance.^92^,^156^,^206^

Autophagy is also a critical pathway for programmed cell death in the cardiovascular system.^114^,^116^,^130^,^199^,^207^,^208^ In the process of autophagy, cytoplasmic organelles and additional subunits are engulfed and recycled for future use.^176^,^177^,^208^,^209^ Three subtypes of autophagy are involved: macroautophagy, microautophagy, and chaperone-mediated autophagy. Macroautophagy is the most common subtype in which autophagosomes are used to merge organelles and cytoplasmic proteins into lysosomes for degradation and recycling. Microautophagy relies upon the invagination of lysosomal membranes to recycle organelles, whereas chaperone-mediated autophagy directly transfers “protein chaperones” to lysosomes for degradation.^145^,^210^,^211^ Autophagy induction can be critical for maintaining metabolic homeostasis in cardiac muscle, since loss of autophagy leads to high-fat diet-induced glucose intolerance, inability to limit oxidative stress during exercise, and reduced muscle endurance.^212^ During DM, the activation of autophagy can protect against cardiac apoptosis and increase cardiac function and tissue structure.^213^ During chronic hypoxia, the activation of autophagy pathways can limit apoptotic myocardial cell death.^214^ During periods of sustained cold stress, autophagy can protect against pressure overload and assist with myocardial contractile capacity.^215^ Autophagy activation is also protective during DM and the loss of cellular glucose homeostasis. Activation of autophagy can reduce diabetic retinopathy,^216^ limit insulin resistance, which can involve growth factors, such as erythropoietin (EPO),^217^,^218^,^219^ protect cardiac cells during DM under conditions of ischemia–reperfusion, control oxidative stress and lipotoxicity, and protect cells during DM-induced cell ischemia through the maintenance of inflammatory cell mitochondrial autophagy activity.^220^

However, autophagy induction is not consistently beneficial and can lead to cell injury during periods of autophagic flux and excessive activation of autophagy.^207^,^221^ Increased autophagy activity may lead to decreased muscle mass,^222^ promote oxidative stress pathways that lead to cell injury,^223^,^224^ disrupt circadian rhythm,^225^,^226^,^227^ promote diabetic retinopathy,^221^ and induce cardiomyocyte cell death during ischemia–reperfusion injury.^228^,^229^ In cases when the modulation of autophagy is essential to protect cell survival, pathways associated with the mechanistic target of rapamycin (mTOR) can complement autophagy pathways that would otherwise lead to injury, since mTOR is an inverse regulator of autophagy. For example, mTOR activation can stabilize atherosclerotic lesions with autophagy inhibition,^230^ can promote growth factor protection, such as with EPO, during oxidative stress for vascular cells,^173^,^231^,^232^,^233^,^234^ and can be necessary for TL stabilization.^235^

## Interdependence between Cardiovascular Disorders, Alzheimer’s Disease, and Cellular Metabolism

Cardiovascular disease has a bidirectional relationship with the nervous system and is associated with cognitive loss and AD.^70^,^107^,^236^,^237^,^238^ Approximately 11% of the population suffers from the sporadic form of AD, which is expected to increase in the US alone from 7 million people to 30 million people by the year 2050.^23^,^24^,^25^,^26^,^239^ In contrast to the sporadic form of AD, which usually affects individuals older than 65 years, familial AD affects approximately 200 families globally before 55 years of age, with amyloid precursor protein (*APP*) gene mutations on chromosomes 1, 14, and 21.^82^,^178^,^184^,^227^,^240^,^241^ MS, which has an increased risk for cardiovascular disease,^242^ is also similar to AD with the loss of cognition, such that memory loss can be progressive with word finding difficulties and object naming impairments.^32^,^34^,^243^,^244^,^245^,^246^

Vascular artery disease of the cardiac system is associated with increased dementia and memory loss,^247^ as well as depression.^248^ In fact, disturbances in behavior can be a precursor or comorbid disease with cardiovascular disease and cognitive loss in AD and MS patients.^27^,^28^,^29^,^30^,^31^,^32^,^33^,^34^,^35^ Almost 25% of individuals with cardiovascular disease, such as angina, congestive heart failure, and myocardial infarction, suffer from depression.^248^ Increased mental health conditions occur in people with developmental and intellectual disability,^249^ and depression is associated with the initial stages of cognitive loss.^250^ Depression and anxiety can trigger inflammation that promotes long-term disability in patients with AD or MS.^30^,^31^,^34^,^251^,^252^ With respect to pathways involved in aging and cell senescence, patients with dilated cardiomyopathy and progressive memory loss have decreased TL length and cell senescence progression.^39^,^157^ In addition, other factors can solidify the link between cardiovascular disease and dementia with AD, such as platelet dysfunction (**Figure 1**). A high-density population of platelets can promote Aβ formation, which leads to AD^253^ and results in oxidative stress with impairment of platelet mitochondria.^254^ The increased presence of atherosclerosis can affect regions of the brain involved in memory, such as the hippocampus,^93^,^255^,^256^ and endothelial dysfunction can lead to neuronal cell injury, altered mitochondrial bioenergetics, and decreased Aβ clearance from the brain.^257^ Microvascular injury in the cardiovascular system and the brain can also result in inflammatory cell injury and facilitate infections such as SARS-CoV-2 and COVID-19.^258^,^259^,^260^

**Figure 1:**
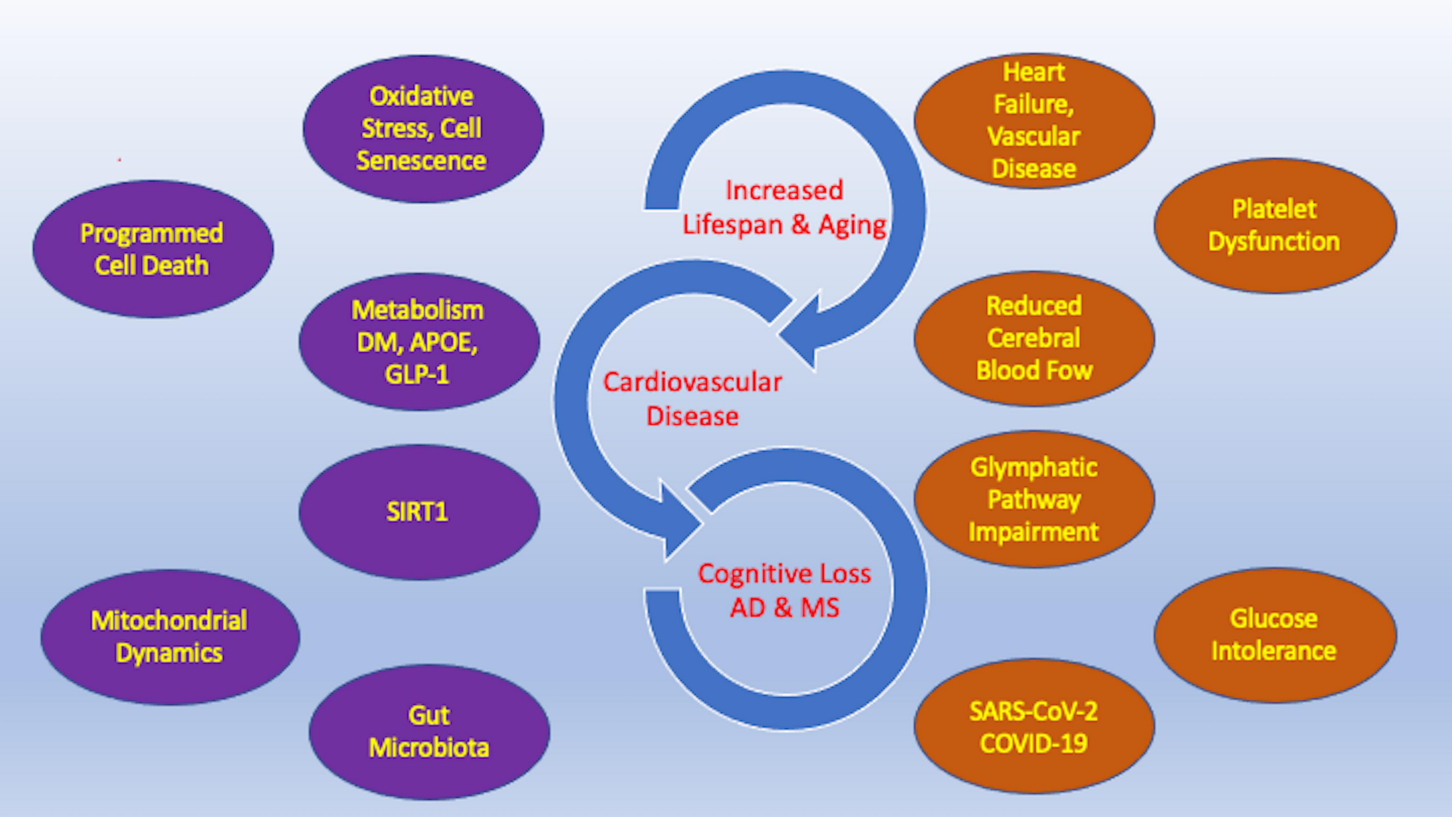
The neurocardiac basis for cardiovascular disease and cognitive loss. Cardiovascular disease and cognitive loss with Alzheimer’s disease (AD) and multiple sclerosis (MS) have a neurocardiac basis that are tied to cellular metabolic pathways that can involve diabetes mellitus (DM). With progressive aging and increased lifespan, non-communicable diseases that involve cardiovascular diseases and metabolic disorders lead to clinical disabilities with heart failure, vascular disease, platelet dysfunction, reduced cerebral blood flow, glymphatic (perivascular) pathway impairment, glucose intolerance, and susceptibility to viral infections with severe acute respiratory syndrome (SARS-CoV-2) and coronavirus disease 2019 (COVID-19) that are directly tied to the loss of cognition, intellectual disability, and behavior health illnesses. Given that therapies for cardiovascular disease, cognitive loss, and co-morbidities with DM are principally symptomatic in nature, innovative avenues that examine the shared underlying pathways of oxidative stress, cellular senescence, programmed cell death with apoptosis, ferroptosis, pyroptosis, and autophagy, cellular metabolism with apolipoprotein E (APOE) and glucagon-like peptide-1 (GLP-1) receptor agonism, the silent mating type information regulation 2 homolog 1 (Saccharomyces cerevisiae) (SIRT1), mitochondrial dynamics, and the gut microbiome are warranted to offer new opportunities to address the shared neurocardiac basis for the onset and progression of cardiovascular and cognitive loss disorders. Created with Microsoft PowerPoint Software (version 16.103.1).

Metabolic disease that involves DM is a critical cellular mechanism that closely links cardiovascular disease to dementia and AD. DM leads to 2 million annual deaths, with half of those deaths occurring in individuals younger than 70 years.^226^,^261^ Many other individuals lack a proper diagnosis and are believed to have prediabetes, with elevated fasting glucose levels and hemoglobin A1c levels.^49^,^166^,^262^ Risk factors for DM include lower education level and socioeconomic status; high blood pressure; elevated serum cholesterol; high alcohol and tobacco consumption; SARS-CoV-2 infection; limited physical activity; and obesity.^33^,^50^,^58^,^261^,^263^,^264^,^265^ In relation to obesity, multiple disorders of metabolism affect both cardiac and cognitive function, including poor cerebral blood flow, oxidative stress, glucose intolerance, insulin resistance, inflammation, impaired stem cell survival, infection susceptibility to COVID-19, and mitochondrial dysfunction.^91^,^118^,^121^,^266^,^267^,^268^,^269^,^270^,^271^,^272^,^273^

During DM, disorders of the cardiovascular system that involve loss of cardiac mitochondrial homeostasis, increased oxidative stress, heightened cardiac inflammation, dyslipidemia, cardiomyocyte apoptosis, impaired cardiac function, and ischemia–reperfusion susceptibility can be multiple factors that foster impaired cognition and AD.^39^,^40^,^41^,^42^,^43^,^44^,^45^,^274^ Both vascular and nervous system components are affected during DM, with systemic autonomic dysfunction resulting in memory loss in AD, vascular dementia, and Parkinson’s disease.^275^,^276^,^277^ Obesity during DM can affect endothelial cell function and increase the permeability of the blood–brain barrier, leading to dementia on a vascular basis.^278^,^279^ The perivascular system is also an important pathway that can be affected by DM and cardiac heart failure with reduced arterial pulse pressure. The perivascular system is a perivascular network of cerebrospinal fluid in the brain that eliminates metabolic waste and toxins such as Aβ, tau, and α-synuclein.^158^,^280^,^281^,^282^ This system can fail during periods of sleep loss and sleep fragmentation involving circadian rhythm impairment, resulting in cardiovascular dysfunction, inflammation, and cognitive loss with long-COVID-19 infection, also known as long-haul COVID-19, chronic COVID-19, or post-acute COVID-19.^57^,^58^,^109^,^110^,^166^,^283^,^284^,^285^,^286^,^287^ During DM, perivascular system function may be significantly impaired, which can add to prior dysfunction from cardiac insufficiency.^158^,^288^ With cardiac impairment during DM, the loss of metabolic homeostasis can also result in Aβ and tau deposition, increased Aβ and tau toxicity with insulin resistance, and the activation of programmed cell death pathways with autophagy.^33^,^96^,^289^,^290^,^291^,^292^ As demonstrated by elevated serum levels of neurofilament light chain, which can be used as an early diagnostic marker of brain cell death, long-term injury to the cortex of the brain can lead to the development of DM.^6^,^293^,^294^

DM can also lead to a decrease in the expression of growth factors, such as EPO, that can impair both cardiac and neurological function. EPO in the cardiovascular system is necessary to prevent cardiac fibrosis and microvessel disease,^295^ promotes positive ionotropic effects,^296^ limits hypoxic cardiac cell death,^297^,^298^ and stimulates coronary collateral development.^299^ In the nervous system, EPO can limit inflammatory cell responses,^300^ improve verbal memory and reduce behavior disorders,^301^ protect endothelial cells,^117^,^173^,^302^,^303^ reduce injury from ischemic environments,^304^,^305^ and assist with nerve myelination.^306^ EPO also functions as a modulatory regulator of autophagy, since EPO can rely upon mTOR pathways^117^,^182^,^307^,^308^ and decrease the activity of autophagy to promote cell survival.^309^

With respect to cellular metabolism pathways, APOE is a critical component that plays a significant role in cardiovascular disorders and neurodegenerative diseases, including AD. APOE is necessary for metabolism to oversee lipid function and the transport of phospholipids, triglycerides, and cholesterol.^33^,^58^,^166^,^310^,^311^,^312^,^313^ Peripherally, APOE is produced by liver cells, but in the nervous system, astrocytes produce APOE to transfer cholesterol to neurons through receptors of APOE.^83^,^139^ In the cardiovascular system, the ε4 allele of the APOE (*APOE-ε4*) gene can be an important risk factor for heart disease and early-onset cardiovascular dysfunction, whereas *APOE-ε2* can be related to elevated triglyceride levels, and *APOE-ε3* is not thought to lead to increased risk for cardiac disease.^314^ In the nervous system, individuals with two *APOE-ε4* alleles (homozygous ε4/ε4) have a 20-fold greater risk of developing AD by the age of 85 years.^166^,^257^,^310^ *APOE-ε4* carrier status, which includes homozygotes and heterozygotes, can also result in accelerated age-related cognitive loss after the age of 70 years.^315^ In individuals with MS, *APOE-ε4* may lead to cognitive impairment, yielding a clinically relevant slower response to stimuli^316^, and the *APOE*-ε3/ε3 genotype may increase the risk for optic neuritis in males.^317^ *APOE-ε4* can also promote viral antigen infection with SARS-CoV-2 and COVID-19, resulting in cognitive loss, microhemorrhages, depression, and long-COVID in AD and MS patients.^34^,^58^,^108^,^318^,^319^ However, *APOE-ε2* alleles have been associated with decreased Aβ aggregation and the prevention of apoptotic PS membrane exposure.^320^

Metabolic disease that links the pathologies of cardiovascular disorders and AD may also center on GLP-1 receptor agonism (**Figure 1**). Currently approved US Food and Drug Administration treatments for obesity and glucose homeostasis, such as the GLP-1 receptor agonists semaglutide, tirzepatide, and liraglutide, may have potential applications throughout the body, such as in cardiovascular and neurological systems.^23^,^118^,^321^,^322^ As G protein-coupled receptors, GLP-1 receptors reside across membranes with an extracellular N-terminus and an intracellular C-terminus and have 7α-helical transmembrane domains. GLP-1 receptors are expressed in pancreatic β-cells, neurons, cardiomyocytes, gastrointestinal cells, renal cells, immune cells, hepatic cells, and cells of the musculoskeletal system.^23^,^322^,^323^,^324^,^325^,^326^ GLP-1 receptor agonism can promote glucose homeostasis, improve insulin receptor function, limit oxidative stress, and prevent mitochondrial dysfunction.^51^,^323^,^324^,^327^,^328^,^329^ The maintenance of glucose homeostasis through GLP-1 receptor agonism may be overseen through the activity of transient receptor potential cation channel subfamily V member 2 (TRPV2) channels.^326^ In this family of channels, the TRPV1 receptor family is a vital contributor to controlling cell survival, cellular metabolism, inflammation, and oxidative stress.^330^,^331^,^332^,^333^ In the cardiovascular system, GLP-1 receptor activation can protect cardiomyocytes and vascular tissue against oxidative stress in patients with DM^329^ and prevent cell injury during doxorubicin exposure.^118^ Similarly, to provide cellular protection in the nervous system, GLP-1 receptor agonism increases neuronal cell survival during experimental models of encephalopathy with DM,^327^ improves cell glucose transport and reduces brain insulin resistance,^51^ functions as a neurotransmitter to improve cognition,^51^ and contributes to altered cellular metabolism in AD,^324^ and it reduces mortality with brain metastasis during DM.^334^

GLP-1 receptor agonism can promote mTOR pathway activation to protect pancreatic β-cells against cholesterol-mediated apoptotic cell death and glucose toxicity.^328^,^335^ mTOR with GLP-1 receptor agonism is also essential for protection during cognitive loss and depression, which can occur with the loss of metabolic homeostasis.^336^ GLP-1 receptor activation also requires mTOR activity and autophagy modulation to promote neuronal survival in models of Huntington’s disease and encephalopathy with DM.^327^,^337^ However, under some circumstances, GLP-1 receptor activation requires the induction of autophagy with mTOR inhibition to induce retinopathy during DM216 and during cellular protection in osteoarthritis.^338^ The modulation of autophagic flux is vital for the use of GLP-1 receptors in both cardiac and cognitive loss disorders and may be related to the emerging “off-target” clinical effects of GLP-1 receptor agonists, which include anterior ischemic optic neuropathy, acute pancreatitis, depression, alopecia, nausea, vomiting, and diarrhea.^339^,^340^,^341^,^342^

## Influence of SIRT1, Mitochondrial Dynamics, and the Gut Microbiota

Given the role cellular metabolism plays in cardiovascular disease and AD, the SIRT1 pathway is a critical mechanism that links cardiovascular disease with neurodegeneration.^39^,^43^,^62^,^114^,^115^,^134^,^167^,^343^,^344^,^345^,^346^,^347^ Seven mammalian homologs of Sir2, including SIRT1, SIRT2, SIRT3, SIRT4, SIRT5, SIRT6, and SIRT7, oversee aging processes, cell development and proliferation, oxidative stress, cellular senescence, cellular metabolism, and post-translational protein modifications.^39^,^348^,^349^,^350^,^351^,^352^,^353^,^354^,^355^,^356^,^357^,^358^,^359^ In regard to SIRT1, this enzyme is a histone deacetylase that oversees the transcription of DNA through acetyl group transfer from ε-N-acetyl lysine amino acids to DNA histones and is present in the liver, heart, brain, skeletal muscle, spleen, pancreas, and adipose tissue.^116^,^132^,^287^,^360^,^361^,^362^,^363^ NAD^+^ is a substrate for SIRT1.^18^,^123^,^226^,^362^,^364^,^365^,^366^ SIRT1 controls nicotinamide phosphoribosyl-transferase, which is necessary for NAD^+^ production. NAD^+^ is crucial for blocking vascular disease and dementia and maintaining mitochondrial function.^90^,^292^,^365^,^367^ Fluctuations in pools of cellular NAD^+^ can occur with aging and changes in circadian clock gene expression.^7^,^143^,^149^,^368^

Interestingly, the agent nicotinamide, the amide form of vitamin B3 (niacin), is a precursor for NAD^+^ and functions to reduce oxidative stress, increase cell survival during apoptosis, maintain mitochondrial membrane potential, reduce inflammation, and control insulin resistance.^11^,^122^,^369^,^370^,^371^,^372^,^373^,^374^ SIRT1 and nicotinamide are dependent upon one another through NAD^+^.^107^,^165^,^258^,^347^,^375^ Nicotinamide can be produced by SIRT1, promoting the transfer of the acetyl residue of the histone acetyllysine residue to the ADP-ribose moiety of NAD^+^. Cellular protection with nicotinamide can occur in a narrow concentration range, since high levels can limit SIRT1 protection by binding nicotinamide to sirtuins through NAD^+^ in the sirtuin C pocket and noncompetitively inhibiting SIRT1.^376^ As a feedback mechanism, SIRT1 activation through nicotinamide phosphoribosyl-transferase increases NAD^+^ levels and decreases nicotinamide levels such that they become insufficient to block SIRT1 activity.^377^ Increased physical activity can facilitate this process.^368^ In this feedback pathway, nicotinamide can also limit SIRT1 activity through the interception of an ADP-ribosyl-enzyme-acetyl peptide intermediate with the regeneration of NAD^+^,^378^ which may be necessary at times to restore acetylation levels, mitigate mitochondrial impairment, and limit autophagy.^362^,^379^

In the cardiovascular system, SIRT1 oversees multiple cardiac disease entities, including hypertension, atherosclerosis, cardiac failure, and myocardial function (**Figure 1**).^43^,^112^,^113^,^380^ Patients with inflammatory dilated cardiac myopathy have increased inflammatory markers, especially in women, when compared with men, but lack vital elevations in SIRT1 activity, suggesting a failure for sufficient SIRT1 activity to offer cytoprotective pathways for these patients.^39^ In experimental models of DM cardiomyopathy and cardiac hypertrophy, SIRT1 activation is necessary to reduce oxidative stress, block apoptosis, and maintain mitochondrial function.^114^,^120^,^134^ In models of cardiotoxicity and aging, SIRT1 can promote cellular proliferation, limit programmed cell death, reduce inflammation, and diminish oxidative stress.^70^,^115^,^118^,^167^ During periods of lipotoxicity and the loss of metabolic homeostasis, SIRT1 can restore physiological cellular metabolism through increased physical activity.^44^,^78^,^381^ Increased expression of SIRT1 can reduce cellular senescence, improve cardiac performance, promote stem cell viability, and limit endothelial cell dysfunction during myocardial infarction.^43^,^78^,^382^,^383^,^384^ SIRT1 activation through heightened NAD^+^ can reduce cardiac injury, limit inflammation, and promote metabolic homeostasis.^385^,^386^,^387^,^388^ Growth factors, such as EPO, also rely upon SIRT1 activation to maintain cellular function, such as during exposure to toxic agents and oxidative stress.^218^,^233^,^389^,^390^

Considering its role in cognitive loss and metabolic dysfunction, SIRT1 is associated with DM and can function as a regulatory protein for glucose homeostasis and obesity in several clinical populations.^391^,^392^,^393^ SIRT1 can reduce oxidative stress, restore glucose homeostasis and insulin levels, and improve cellular survival in experimental models of DM.^40^,^89^,^114^,^216^,^268^,^394^ In models of cognitive loss and AD, SIRT1 can mitigate Aβ toxicity and preserve cellular survival.^343^,^344^,^395^ SIRT1 activation can decrease oxidative stress in the brain, block apoptosis, improve spatial memory, and increase synaptic density.^345^,^346^,^347^,^396^,^397^ SIRT1 can promote neuronal stem cell differentiation, oversee the microglial inflammatory response, maintain astrocyte survival, and prevent blood–brain barrier disruption.^373^,^398^,^399^,^400^,^401^ Through the activation of SIRT1, the memory function of hippocampal neurons is maintained, synaptic function is preserved, and the clearance of tau and Aβ is promoted in models of AD.^361^,^402^,^403^,^404^,^405^,^406^ A lack of SIRT1 activity may lead to inflammation, delays in wound healing, and neurodegeneration with decreased lifespan.^407^,^408^,^409^

During cardiovascular disease and dementia, mitochondrial dynamics also play a significant role in the onset and progression of these disorders through pathways that involve cellular metabolism. In patients with DM, mitochondrial dysfunction can be associated with impaired energy expenditure, loss of cardiac contractility, renal impairments, ferroptosis activation, apoptotic cell death, oxidative stress, insulin resistance, circadian rhythm disorders, altered lipid metabolism, endothelial cell loss, and pancreatic β-cell loss.^49^,^114^,^156^,^261^,^267^,^410^,^411^,^412^,^413^,^414^,^415^ Mitochondrial integrity is necessary for redox cellular signaling, which is involved in physiological metabolic consumption.^163^,^416^ Loss of mitochondrial function results in caspase activation, endoplasmic reticulum stress, microvascular disease, programmed cell death, and mitoptosis.^25^,^77^,^131^,^158^,^164^,^165^,^346^,^351^,^417^,^418^,^419^,^420^

SIRT1 significantly affects the maintenance of mitochondrial dynamics during cardiovascular and memory loss disorders.^62^,^362^,^380^,^421^ During cardiac aging, SIRT1 maintains mitochondrial integrity to block inflammation and oxidative stress in cardiac tissue.^70^,^167^ During oxidative stress, SIRT1 can modulate autophagy and mitochondrial function in embryonic stem cells,^422^ reduce severe cardiac dysfunction through SIRT1 activity and the maintenance of mitochondrial integrity,^44^,^384^ and protect coronary artery endothelial cells from mitochondrial oxidative stress.^423^ In experimental models of AD, SIRT1 can block Aβ cell toxicity, preserve mitochondrial membrane potential, reduce the generation of ROS,^343^ and prevent apoptotic cell death.^346^,^424^ In the absence of SIRT1 and the loss of mitochondrial integrity, Aβ toxicity, memory loss, and programmed cell death of microglia, neurons, and astrocytes can occur.^404^,^425^,^426^ Intact cognition also appears to be reliant upon GLP-1 activation with SIRT1 and mitochondrial preservation, which can protect both cardiac and neurological function.^118^,^337^

In addition to cellular metabolism pathways, SIRT1, and mitochondrial dynamics, one of the important complex ecosystems of the body that can link cardiovascular disease and AD is the gut microbiota (**Figure 1**).^414^,^427^,^428^,^429^,^430^ Approximately 100 billion bacteria form the gut microbiota, and this system functions as an endocrine organ since beneficial organisms can generate short-chain fatty acids and synthesize vitamins such as vitamin B for the host to consume throughout the body.^155^,^239^,^431^,^432^ The gut microbiota is connected to protective pathways for both the cardiovascular system and the nervous system that involve SIRT1 activity. Experimental studies that clarify Aβ and tau aggregates for the treatment of AD rely upon SIRT1 activation and the gut microbiota to regulate indole metabolites, such as 3-indollepropionic acid, which may be neuroprotective.^30^,^361^ Investigations have shown that altering the gut microbiota to promote SIRT1 activation and suppress inflammatory cytokines leads to improved memory and blocks degeneration of the hippocampus in models of AD.^403^ Increased SIRT1 expression can also protect the vascular endothelium, improve cardiovascular function, and block inflammation during high-fat diets through the prevention of detrimental effects on the gut microbiota that normally impair vascular endothelial function.^433^,^434^ Recent investigations have employed directed changes in the gut microbiota, such as to reduce the abundance of Bacteroidota and Firmicutes species, that offer beneficial effects in AD models with aging and assist with cardiovascular disorders to improve memory, prevent hippocampal neuronal cell loss, and promote cardiac function.^427^,^430^,^435^,^436^,^437^,^438^ Other studies of the gut microbiota in individuals with DM also point to increasing species, such as Lactobacillus, with mTOR activation, which can increase the levels of short-chain fatty acids to reduce inflammation, block insulin resistance, and improve metabolic homeostasis to improve cardiovascular and neurological systems.^92^,^155^,^432^,^439^

## Limitations

Examinations of the neurocardiac basis related to cardiovascular disease and cognitive loss that investigate biological gas pathways are considered innovative and represent a developing line of investigations. As a result, there exist limitations and challenges to be recognized. First, the role of biological gas pathways related to oxidative stress and the presence of ROS is not simplistic in nature and is tied to multiple cellular pathways that can influence clinical outcomes. The generation of ROS can lead to cell injury in tissue- and cell-specific regions that is overseen by multiple factors that can include whether inflammatory cell activation is beneficial or detrimental, whether cellular energy pathways are maintained at physiological levels, and whether protective pathways that can include SIRT1 are sufficiently effective during redox energy balance that can be sex specific. Second, our knowledge of metabolic pathways is under continual growth and requires further clarification. For example, *APOE-ε4* gene is a significant risk factor for cardiac disease, early-onset cardiovascular dysfunction, accelerated age-related cognitive loss, and viral infection with SARS-CoV-2 and COVID-19, leading to brain microhemorrhages and mental disability. However, other subtypes of APOE can affect specific tissues and systems of the body, with *APOE-ε2* related to elevated triglyceride levels in the cardiovascular system, decreased brain Aβ aggregation and a reduction in apoptotic membrane PS exposure in the nervous system, and *APOE-ε3* is either not detrimental to cardiac disease or potentially affects only males with optic neuritis in the nervous system. Third, the shared pathways bridging cardiovascular disease with cognitive loss are at early developmental stages for clinical and diagnostic use. Therefore, additional studies will be critical for clinical translation to human care from the present *in vitro* and *in vivo* animal models. Future clinical trials will be necessary to help determine whether specific subsets of cardiovascular disease, such as individuals with atherosclerosis or heart failure, share underlying pathways that can also be targeted for either early- or late-stage cognitive loss and AD. Even current clinical studies require further development with new trials that consider patient comorbidities that can affect clinical outcomes, as evidenced by work with new diagnostic methods, such as neurofilament light chains, that can detect early brain neuronal cell death in patients with cognitive loss but may be valid only in a limited subset of patients. These limitations elucidate the necessary path forward to translate into clinical care the shared underlying pathways for neurocardiac care.

## Conclusion and Future Perspectives

In both developed and developing nations, lifespan is significantly increasing, with the number of individuals over the age of 65 doubling during the prior 50 years, and over the next two decades, it is expected that more than 2 billion people will reach a lifespan of 80 years old. With the increase in lifespan there has been a corresponding rise in NCDs. Cardiovascular disease representes the leading cause of death annually, and a significant portion of the global population is suffering from cognitive loss and AD. Interestingly, cardiovascular disease and cognitive loss with AD form a neurocardiac axis by sharing underlying cellular mechanisms that can lead to the onset and progression of these disorders. Approximately one-quarter of individuals with cardiovascular disease that involves angina, congestive heart failure, and myocardial infarction suffer from changes in cognition because of platelet dysfunction, atherosclerosis, endothelial disease, metabolic disease with DM, perivascular pathway failure, and microvascular injury. These disorders lead to the progression of cognitive loss and AD with decreased brain Aβ and tau clearance and susceptibility to long-COVID. Metabolic pathways tied to APOE also play a significant role in cardiovascular disease and AD with *APOE-ε4* carrier status, resulting in early-onset cardiovascular dysfunction, accelerated age-related cognitive loss, and memory loss with viral infection of SARS-CoV-2 and long-COVID-19. Given that current therapies for cardiovascular disease, cognitive loss with AD, and comorbidities with DM are primarily symptomatic in nature, innovative strategies that examine the shared underlying cellular mechanisms of these disorders are highly warranted for the development of future clinical care. Investigations into oxidative stress, cellular senescence, programmed cell death with apoptosis, ferroptosis, pyroptosis, and autophagy, cellular metabolism with APOE and GLP-1 receptor agonism, SIRT1, mitochondrial dynamics, and the gut microbiome can offer new and invaluable insights into effective strategies that can address risk factors and clinical treatments for cardiovascular disease and AD (**Figure 1**). Notably, these pathways have intimate and complex relationships that necessitate thoughtful approaches. For instance, although autophagy induction may at times suppress oxidative stress and enhance cell survival in the cardiovascular and neurological systems, pathways such as those involving mTOR can become essential, particularly during aging via TL-related processes and through directed modulation of the gut microbiota, to regulate and oversee dysfunctional autophagic flux that might otherwise block the desired clinical outcomes of agents such as GLP-1 receptor agonists and growth factors like EPO.

**Facts**

1. Cardiovascular disease, Alzheimer’s disease, and multiple sclerosis share a neurocardiac basis linked by cellular metabolism and diabetes, indicating the presence of common pathological pathways.
2. Current care remains symptomatic and prevention focused, and interventions targeting shared pathways such as oxidative stress, senescence, and autophagy are lacking.
3. Apolipoprotein E (APOE), glucagon-like peptide-1 (GLP-1) agonism, silent mating type information regulation 2 homolog 1 (*Saccharomyces cerevisiae*) (SIRT1), mitochondrial dynamics, and the gut microbiome are key modulators of the neurocardiac axis and are highly interdependent.
4. Understanding the interactions among ferroptosis, pyroptosis, and apoptosis under comorbid conditions is essential for clinical translation.

**Open Questions**

1. Is oxidative stress a primary driver or a consequence of autophagy dysregulation that links cardiac dysfunction to cognitive decline?
2. Are GLP-1 receptor agonists neuroprotective independent of glycemic control, and what is the optimal timing and disease stage?
3. Which microbial metabolites influence mitochondrial dynamics and senescence, and can microbiome targeting improve both cardiac and cognitive outcomes?
4. Is combined inhibition of ferroptosis and pyroptosis superior to single-pathway blockade, and how can strategies for different comorbidity profiles be chosen?
5. How can *APOE* genotypes and individual metabolic states guide personalized therapies that simultaneously reduce cardiovascular risk and neurodegeneration?

## Data Availability

*Not available.*

## References

[1] Sutherland M, Biswell E, Raidal S (2026). Recent advances in cancer diagnostics: exotic animal medicine and beyond. Vet Clin North Am Exot Anim Pract.

[2] Zhao LX, Wang ET, Jia WH (2026). Emerging salivary exosome nanodiagnostics for early oral squamous cell carcinoma: platforms, biomarkers, and future perspectives. Int J Nanomedicine.

[3] Elias MG, Hadjiyiannis H, Vafaee F (2025). The quest for non-invasive diagnosis: a review of liquid biopsy in glioblastoma. Cancers (Basel).

[4] Jin D, Khan NU, Gu W, Lei H, Goel A, Chen T (2025). Informatics strategies for early detection and risk mitigation in pancreatic cancer patients. Neoplasia.

[5] Daponte A, Koros C, Skarlis C (2025). Neurofilament biomarkers in neurology: from neuroinflammation to neurodegeneration, bridging established and novel analytical advances with clinical practice. Int J Mol Sci.

[6] Foser S, Maiese K, Digumarthy SR, Puig-Butille JA, Rebhan C (2024). Looking to the future of early detection in cancer: liquid biopsies, imaging, and artificial intelligence. Clin Chem.

[7] Maiese K (2023). Cornerstone cellular pathways for metabolic disorders and diabetes mellitus: non-coding RNAs, Wnt signaling, and AMPK. Cells.

[8] Yang B, Li A, Mohib AH (2025). Tongue image analysis for accurate prediction of nutritional risk screening in cancer patients during radiochemotherapy: a feature selection network and aliasing attention mechanism approach. Front Nutr.

[9] Kumar P, Pal Y, Singh SD, Tiwari SB (2025). A comprehensive review on cardiovascular and chronic kidney disease interplay, exploring biomarkers and algorithmic approaches in unraveling organ crosstalk. Indian J Pharmacol.

[10] Li M, Yang Y, Wang Z (2021). Piwi-interacting RNAs (piRNAs) as potential biomarkers and therapeutic targets for cardiovascular diseases. Angiogenesis.

[11] Maiese K (2020). New Insights for nicotinamide: Metabolic disease, autophagy, and mTOR. Front Biosci (Landmark Ed).

[12] Quotah OF, Poston L, Flynn AC, White SL (2022). Metabolic profiling of pregnant women with obesity: an exploratory study in women at greater risk of gestational diabetes. Metabolites.

[13] Zaiou M (2020). circRNAs signature as potential diagnostic and prognostic biomarker for diabetes mellitus and related cardiovascular complications. Cells.

[14] Zhu Y, Fang Q, Zhou Y, Lu W, Du X, Shi B (2025). Serum Wnt1-inducible signalling pathway Protein-1 levels are associated with cerebral infarction in patients with type 2 diabetes mellitus. J Endocrinol Invest.

[15] de Cavanagh EMV, Inserra F, Ferder L (2024). Renin-angiotensin system inhibitors positively impact on multiple aging regulatory pathways: Could they be used to protect against human aging?. Physiol Rep.

[16] Lathe R, St Clair D (2023). Programmed ageing: decline of stem cell renewal, immunosenescence, and Alzheimer’s disease. Biol Rev Camb Philos Soc.

[17] Wilson SJ (2024). Is age more than a number? Accounting for adult development and aging in the study of psychoneuroimmunology, stress, and health. Comprehensive psychoneuroendocrinology.

[18] Yadav S, Pan X, Li S (2026). Perturbation of NAD(P)H metabolism with the LbNOX xenotopic tool extends lifespan and mitigates age-related changes. Sci Adv.

[19] Ronan G, Hawthorne L, Yang J (2026). “Comprehensive multi-omics of age-respective plasma and matrix-bound extracellular vesicles identifies anti-fibrotic miRNAs validated on a heart-on-a-chip”. Biomaterials.

[20] Rotllan N, Camacho M, Tondo M (2021). Therapeutic potential of emerging NAD+-increasing strategies for cardiovascular diseases. Antioxidants (Basel).

[21] Maiese K, Chong ZZ, Shang YC, Hou J (2009). FoxO proteins: cunning concepts and considerations for the cardiovascular system. Clin Sci (Lond).

[22] Stefani M, Rigacci S (2014). Beneficial properties of natural phenols: highlight on protection against pathological conditions associated with amyloid aggregation. Biofactors.

[23] Maiese K (2026). Cannabis and cannabidiol, GLP-1 receptors, and autophagy: the burgeoning link between cognitive neurodegeneration with Alzheimer’s disease and metabolic disorders. Discov Med.

[24] Soni S, Kaur G (2026). Emerging therapeutics targeting the PI3K/Akt/GSK-3β in calcium dysregulation in Alzheimer’s disease. Cell Calcium.

[25] Sultana OF, Bandaru M, Bushra MA, Reddy PH, Reddy AP (2026). Serotonin-mediated regulation of mitophagy in Alzheimer’s disease: mechanistic insights and therapeutic potential. Ageing Res Rev.

[26] Yip KC, Ho WF, Liu Y, Dawe GS (2026). APP-C31 pathology as a target in neurodegenerative diseases. J Biomed Sci.

[27] Amidfar M, Garcez ML, Kim YK (2023). The shared molecular mechanisms underlying aging of the brain, major depressive disorder, and Alzheimer’s disease: the role of circadian rhythm disturbances. Prog Neuropsychopharmacol Biol Psychiatry.

[28] Baskarapantula SS, Kumar VS, Changdar P (2025). The 4E-BPs as translational regulators in neurological disorders: molecular mechanisms and therapeutic potential. Mol Neurobiol.

[29] Fan X, Zhao Z, Wang D, Xiao J (2020). Glycogen synthase kinase-3 as a key regulator of cognitive function. Acta Biochim Biophys Sin (Shanghai).

[30] Shafiek MS, Mekky RY, Nassar NN, El-Yamany MF, Rabie MA (2024). Vortioxetine ameliorates experimental autoimmune encephalomyelitis model of multiple sclerosis in mice via activation of PI3K/Akt/CREB/BDNF cascade and modulation of serotonergic pathway signaling. Eur J Pharmacol.

[31] Su LD, Wang N, Han J, Shen Y (2022). Group 1 metabotropic glutamate receptors in neurological and psychiatric diseases: mechanisms and prospective. Neuroscientist.

[32] Sedighi B, Afshar PJ, Jafari S (2025). Investigating the associated factors of mood disorders and fatigue in multiple sclerosis patients with cognitive impairment: a cross-sectional study. Health Sci Rep.

[33] Maiese K (2023). The metabolic basis for nervous system dysfunction in Alzheimer’s disease, Parkinson’s disease, and Huntington’s disease. Curr Neurovasc Res.

[34] Maiese K (2023). Cognitive impairment in multiple sclerosis. Bioengineering (Basel).

[35] COVID-19 Mental Disorders Collaborators (2021). Global prevalence and burden of depressive and anxiety disorders in 204 countries and territories in 2020 due to the COVID-19 pandemic. Lancet.

[36] Torii T, Miyamoto Y, Nakata R (2023). Identification of tau protein as a novel marker for maturation and pathological changes of oligodendrocytes. Glia.

[37] Tiu VE, Popescu BO, Enache, II, Tiu C, Terecoasa E, Panea CA (2022). Serum and CSF biomarkers predict active early cognitive decline rather than established cognitive impairment at the moment of RRMS diagnosis. Diagnostics (Basel).

[38] LaCroix MS, Mirbaha H, Shang P (2022). Tau seeding in cases of multiple sclerosis. Acta Neuropathol Commun.

[39] Barcena ML, Tonini G, Haritonow N (2023). Sex and age differences in AMPK phosphorylation, mitochondrial homeostasis, and inflammation in hearts from inflammatory cardiomyopathy patients. Aging Cell.

[40] Hajibabaie F, Abedpoor N, Safavi K, Taghian F (2022). Natural remedies medicine derived from flaxseed (secoisolariciresinol diglucoside, lignans, and α-linolenic acid) improve network targeting efficiency of diabetic heart conditions based on computational chemistry techniques and pharmacophore modeling. J Food Biochem.

[41] Li Z, Wang B, Bai D, Zhang L (2024). Brazil nut (Bertholletia excelsa) and metformin abrogate cardiac complication in fructose/STZ-induced type 2 diabetic rats by attenuating oxidative stress and modulating the MAPK-mTOR/NFkB/IL-10 signaling pathways. Food Nutr Res.

[42] Loesch DP, Garg M, Matelska D (2025). Identification of plasma proteomic markers underlying polygenic risk of type 2 diabetes and related comorbidities. Nat Commun.

[43] Maiese K (2024). Cardiovascular and nonalcoholic fatty liver disease: sharing common ground through SIRT1 pathways. World J Cardiol.

[44] Rami M, Rahdar S, Aghili Nasab H, Adelnia R, Rajizadeh MA (2025). High-intensity interval training (HIIT) ameliorates cardiac hypertrophy and fibrosis in diabetic rats: the role of P53 and SIRT1. J Mol Histol.

[45] Xia W, Xie X, Wang C (2025). Reduction in cardiac STAT3 phosphorylation at site Ser-727 subsequent to mTOR overactivation exacerbated myocardial ischemia reperfusion injury in type 1 diabetic rats. Fortune J Health Sci.

[46] Maiese K (2012). The many facets of cell injury: angiogenesis to autophagy. Curr Neurovasc Res.

[47] Din ZU, Xia J, Wu ZG, Liao Z, Xiong XD (2025). Circular RNAs and endothelial dysfunction: mechanistic crosstalk and emerging therapeutic perspectives. Cell Mol Life Sci.

[48] Schiano C, Benincasa G, Franzese M (2020). Epigenetic-sensitive pathways in personalized therapy of major cardiovascular diseases. Pharmacol Ther.

[49] Maiese K (2023). The impact of aging and oxidative stress in metabolic and nervous system disorders: programmed cell death and molecular signal transduction crosstalk. Front Immunol.

[50] Satyam SM, Prabhakar S, El-Tanani M (2026). Chronopharmacology-driven precision therapies for time-optimized cardiometabolic disease management. Biology (Basel).

[51] Giofrè F, Zaffina I, Pelle MC, Arturi F (2025). Recent advances in incretin-based therapy for the treatment of cognitive impairment associated to the type 2 diabetes mellitus: preclinical and clinical studies - a narrative review. Front Endocrinol (Lausanne).

[52] Faraji N, Ebadpour N, Abavisani M, Gorji A (2025). Unlocking hope: therapeutic advances and approaches in modulating the Wnt pathway for neurodegenerative diseases. Mol Neurobiol.

[53] Sun R, Xiao Q, Long H (2025). Integrative metabolomics and ionomics identify organ-specific responses associated with cisplatin treatment in mice. Drug Des Devel Ther.

[54] Majumdar S, Samaiya PK, Gupta SK, Krishnamurthy S, Prajapati SK (2026). Aging and neuropathic pain: Mitochondria-to-glia cascade, system mechanisms, and therapeutic strategies. Ageing Res Rev.

[55] Maiese K (2025). Biological gases, oxidative stress, artificial intelligence, and machine learning for neurodegeneration and metabolic disorders. Med Gas Res.

[56] Van de Walle J, Delvaux T, Vandenberghe A (2025). Molecular and cellular drivers of inflammaging: Uncovering the diverse mechanisms and interventions of inflammaging. Clin Transl Rep.

[57] Ishola AA, Ahmed IA, Mikail MA (2026). Molecular roadmap of COVID-19: from viral entry to therapeutic targets. Chem Biodivers.

[58] Tarantini L, Möller C, Schiestl V (2026). Objectifying persistent subjective cognitive impairment following COVID-19 infection: cross-sectional data from an outpatient memory-clinic in Germany. Eur Arch Psychiatry Clin Neurosci.

[59] Anderer S (2026). As US overdose deaths drop, researchers search for explanations. JAMA.

[60] Mathews C (2024). Population ageing in lower- and middle-income countries. celebrating 40 years of helpage international. J Popul Ageing.

[61] Speer H, D’Cunha NM, Alexopoulos NI, McKune AJ, Naumovski N (2020). Anthocyanins and human health-a focus on oxidative stress, inflammation and disease. Antioxidants (Basel).

[62] Maiese K (2021). Targeting the core of neurodegeneration: FoxO, mTOR, and SIRT1. Neural Regen Res.

[63] Saberi F, Dehghan Z, Mehdinejadiani S (2025). Clinical application of cell therapy in the treatment of female reproductive diseases: a systematic review. Cell Reprogram.

[64] Schell M, Wardelmann K, Kleinridders A (2021). Untangling the effect of insulin action on brain mitochondria and metabolism. J Neuroendocrinol.

[65] Chen ST, Chang KS, Hou CP (2026). WISP1 is the stromal-secreting oncoprotein via paracrine downregulation of NDRG1, KAI1, and Maspin in human bladder cancer cells. Transl Oncol.

[66] Wang W, Zhu H, Jiang Q, Shi Y, Wang X (2026). FOXO: a key target in regulating aging and age-related diseases. Biogerontology.

[67] Espinoza C, Veas-Torres J, Miranda DI, Owen GI (2026). Impact of type 2 diabetes on malignancies of the female reproductive system. Mol Aspects Med.

[68] Granata S, Barberio L, D’Agostino R (2026). Emerging therapeutic pipelines on kidney fibrosis: challenges in translational research. J Transl Med.

[69] Talamadla MK, Moharir SR, Katdare M (2026). 2-(1H-Pyrazol-1-yl)pyrimidine derivatives as promising anticancer candidates: synthesis, biological studies, and computational insights. J Mol Struct.

[70] Ju DT, Huang RS, Tsai BC (2023). Folic acid and folinic acid protect hearts of aging triple-transgenic Alzheimer’s disease mice via IGF1R/PI3K/AKT and SIRT1/AMPK pathways. Neurotox Res.

[71] Lee FK, Lee JC, Shui B (2021). Genetically engineered mice for combinatorial cardiovascular optobiology. Elife.

[72] Mehdipour M, Park S, Huang GN (2023). Unlocking cardiomyocyte renewal potential for myocardial regeneration therapy. J Mol Cell Cardiol.

[73] Wang S, Schianchi F, Neumann D (2021). Specific amino acid supplementation rescues the heart from lipid overload-induced insulin resistance and contractile dysfunction by targeting the endosomal mTOR-v-ATPase axis. Mol Metab.

[74] Zhou G, Wu J, Gu C (2018). Prorenin independently causes hypertension and renal and cardiac fibrosis in cyp1a1-prorenin transgenic rats. Clin Sci (Lond).

[75] Castorina A, Szychlinska MA, Marzagalli R, Musumeci G (2015). Mesenchymal stem cells-based therapy as a potential treatment in neurodegenerative disorders: is the escape from senescence an answer?. Neural Regen Res.

[76] Griñán-Ferré C, Vasilopoulou F, Abás S (2019). Behavioral and cognitive improvement induced by novel imidazoline I(2) receptor ligands in female SAMP8 mice. Neurotherapeutics.

[77] Jellinger KA (2024). Cognitive impairment in multiple sclerosis: from phenomenology to neurobiological mechanisms. J Neural Transm (Vienna).

[78] Maiese K (2017). Harnessing the power of SIRT1 and non-coding RNAs in vascular disease. Curr Neurovasc Res.

[79] Mansour MEM, Amin M, Alsaadany KR (2025). Efficacy and safety of pulsed electromagnetic field therapy for fatigue in patients with multiple sclerosis: a systematic review and meta-analysis. Mult Scler Relat Disord.

[80] Mosharaf MP, Alam K, Gow J, Mahumud RA (2023). Exploration of key drug target proteins highlighting their related regulatory molecules, functional pathways and drug candidates associated with delirium: evidence from meta-data analyses. BMC Geriatr.

[81] Oyefeso FA, Muotri AR, Wilson CG, Pecaut MJ (2021). Brain organoids: a promising model to assess oxidative stress-induced central nervous system damage. Dev Neurobiol.

[82] Yaghoobi A, Malekpour SA (2024). Unraveling the genetic architecture of blood unfolded p-53 among non-demented elderlies: novel candidate genes for early Alzheimer’s disease. BMC Genomics.

[83] Caberlotto L, Nguyen TP, Lauria M (2019). Cross-disease analysis of Alzheimer’s disease and type-2 diabetes highlights the role of autophagy in the pathophysiology of two highly comorbid diseases. Sci Rep.

[84] Chen Y, Huang C, Zhu SY, Zou HC, Xu CY, Chen YX (2021). Overexpression of HOTAIR attenuates Pi-induced vascular calcification by inhibiting Wnt/β-catenin through regulating miR-126/Klotho/SIRT1 axis. Mol Cell Biochem.

[85] Debnath A, Ali MA, Deshmukh K, Das A, Shantabi L, Elangbam S (2025). Curcumin as a potential hypoglycaemic agent for managing oxidative stress. Int J Bio-Resour Stress Manage.

[86] Espinoza SE, Khosla S, Baur JA, de Cabo R, Musi N (2023). Drugs targeting mechanisms of aging to delay age-related disease and promote healthspan: proceedings of a national institute on aging workshop. J Gerontol A Biol Sci Med Sci.

[87] Felemban MF, Bin Mubayrik AF, Alotaibi O (2025). The effect of vitamin E, B-carotene, and acidophilus probiotics on the salivary gland flow rate in streptozotocin-induced diabetes in rats: an in-vivo study. Clin Cosmet Investig Dent.

[88] Fischer F, Grigolon G, Benner C, Ristow M (2022). Evolutionarily conserved transcription factors as regulators of longevity and targets for geroprotection. Physiol Rev.

[89] Hadinata E, Harbuwono DS, Soegondo S (2025). Marine nutraceuticals as a source of SIRT1 and NRF2 activators for diabetes and aging-related metabolic disorders. Diabetol Metab Syndr.

[90] Li JJ, Sun WD, Zhu XJ, Mei YZ, Li WS, Li JH (2024). Nicotinamide N-methyltransferase (NNMT): a new hope for treating aging and age-related conditions. Metabolites.

[91] Li Z, Qin N, Ni W (2026). Nanozymes: Reshaping the landscape of age-related disease therapy through multifaceted redox regulation. J Control Release.

[92] Liu Y, Li K, Xu J (2025). Alpha-linolenic acid ameliorates T2DM via reshaping gut-liver axis and inflammatory GPR120-NF-κB/NLRP3 pathway in mouse and rat models. Phytomedicine.

[93] Maiese K (2018). Novel treatment strategies for the nervous system: circadian clock genes, non-coding RNAs, and forkhead transcription factors. Curr Neurovasc Res.

[94] Maiese K, Chong ZZ, Shang YC (2008). OutFOXOing disease and disability: the therapeutic potential of targeting FoxO proteins. Trends Mol Med.

[95] Nguyen BL, Kehmeier MN, Babcock MC (2026). Endothelial sirtuins and mitochondrial function are associated with testosterone status: implications for accelerated vascular aging in middle-age and older men with low testosterone. Aging Cell.

[96] Pattanaik SK, Anil PM, Jena S, Rath D (2025). Interlinking diabetes and Alzheimer’s disease: a pathway through medicinal plant-based treatments. J Ethnopharmacol.

[97] Zhang Y, Yuan Y, Zhang J, Zhao Y, Zhang Y, Fu J (2022). Astragaloside IV supplementation attenuates cognitive impairment by inhibiting neuroinflammation and oxidative stress in type 2 diabetic mice. Front Aging Neurosci.

[98] Abella BS, Alvarado JP, Myklebust H (2005). Quality of cardiopulmonary resuscitation during in-hospital cardiac arrest. JAMA.

[99] Lee YS, Choi JR, Kim JB (2024). Gene therapy for cardiovascular disease: clinical perspectives. Yonsei Med J.

[100] Maiese K (2020). Dysregulation of metabolic flexibility: The impact of mTOR on autophagy in neurodegenerative disease. Int Rev Neurobiol.

[101] Heron M (2019). Deaths: leading causes for 2017. Natl Vital Stat Rep.

[102] Freedman AA, Colangelo LA, Ning H (2026). Sex differences in age of onset of premature cardiovascular disease and subtypes: the coronary artery risk development in young adults study. J Am Heart Assoc.

[103] Bi SZ, Sun WD, Zhu XJ (2025). Nicotinamide N-methyltransferase in cardiovascular diseases: mechanistic insights and therapeutic potential. Eur J Med Chem.

[104] du Toit WL, Kruger R, Gafane-Matemane LF, Schutte AE, Louw R, Mels CMC (2022). Urinary metabolomics profiling by cardiovascular risk factors in young adults: the African prospective study on early detection and identification of cardiovascular disease and hypertension study. J Hypertens.

[105] du Toit WL, Kruger R, Gafane-Matemane LF, Schutte AE, Louw R, Mels CMC (2023). Markers of arterial stiffness and urinary metabolomics in young adults with early cardiovascular risk: the African-PREDICT study. Metabolomics.

[106] Margrett JA, Schofield T, Martin P (2022). Novel functional, health, and genetic determinants of cognitive terminal decline: Kuakini Honolulu Heart Program/Honolulu-Asia Aging study. J Gerontol A Biol Sci Med Sci.

[107] Maiese K (2015). SIRT1 and stem cells: In the forefront with cardiovascular disease, neurodegeneration and cancer. World J Stem Cells.

[108] Chen F, Ke Q, Wei W, Cui L, Wang Y (2023). Apolipoprotein E and viral infection: risks and mechanisms. Mol Ther Nucleic Acids.

[109] Maiese K (2020). The mechanistic target of rapamycin (mTOR): novel considerations as an antiviral treatment. Curr Neurovasc Res.

[110] Shokri-Afra H, Saber Jeyvan F, Barartabar Z (2025). Targeting SIRT1: a potential strategy for combating severe COVID-19. Biomed Res Int.

[111] Kazi DS, Elkind MSV, Deutsch A (2024). Forecasting the economic burden of cardiovascular disease and stroke in the United States through 2050: a presidential advisory from the American Heart Association. Circulation.

[112] Maiese K (2023). Innovative therapeutic strategies for cardiovascular disease. EXCLI J.

[113] Dinislam K, Khoso MA, Kataev VA (2025). SIRT1: the first key to unlocking the mystery of cardiovascular diseases. Front Pharmacol.

[114] Fu R, Hao J (2025). Resveratrol mitigates diabetes-induced cardiac dysfunction via SIRT1/PPAR-α/PGC-1 pathway. Mol Genet Genomics.

[115] Guo L, Yang B, Chen F (2026). Disruption of the SIRT1/PI3K/AKT signaling axis mediates fluoride-induced cardiotoxicity: evidence from in vitro and zebrafish models. Biol Trace Elem Res.

[116] Wen H, Hun M, He Q (2025). Histone deacetylases in myocardial infarction: Orchestrating programmed cell death pathways and emerging therapeutic opportunities. Biomed Pharmacother.

[117] Maiese K (2015). mTOR: Driving apoptosis and autophagy for neurocardiac complications of diabetes mellitus. World J Diabetes.

[118] Salmanoglu M, Ercan G, Genç HS, Gül SS, Aygün H (2026). GLP-1 receptor agonist exenatide protects against doxorubicin-induced cardiotoxicity through the SIRT1 pathway: an electrocardiographic, 99mTc-PYP scintigraphic, and biochemical study. Medicina (Kaunas).

[119] Ponzetti M, Rucci N, Falone S (2023). RNA methylation and cellular response to oxidative stress-promoting anticancer agents. Cell Cycle.

[120] Ramadhan AY, Soetikno V (2024). Molecular adaptation of cardiac remodeling in metabolic syndrome: focus on AMPK, SIRT1 and PGC-1a. Mol Cell Biomed Sci.

[121] Ravi PS, Babu S (2025). The mechanistic interplay of reactive oxygen species in metabolic pathways and insulin signaling in type 2 diabetes. Mol Biol Rep.

[122] Giacalone S, Spigariolo CB, Bortoluzzi P, Nazzaro G (2021). Oral nicotinamide: the role in skin cancer chemoprevention. Dermatol Ther.

[123] Goulart Nacácio ESS, Occhiutto ML, Costa VP (2023). The use of Nicotinamide and Nicotinamide riboside as an adjunct therapy in the treatment of glaucoma. Eur J Ophthalmol.

[124] Joshi SM, Thomas TC, Jadavji NM (2024). Impact of increasing one-carbon metabolites on traumatic brain injury outcome using pre-clinical models. Neural Regen Res.

[125] Musa M, Zeppieri M, Atuanya GN (2023). Nutritional factors: benefits in glaucoma and ophthalmologic pathologies. Life (Basel).

[126] Parab S, Parekh N, Apte K, Shah AK, Tappia PS, Dhalla NS (2024). Unraveling the mechanisms of hydrophilic vitamins in Alzheimer’s and Parkinson’s: preclinical and clinical evidence. Hydrophilic vitamins in health and disease.

[127] Raghuvanshi DS, Chakole S, Kumar M (2023). Relationship between vitamins and diabetes. Cureus.

[128] Zhou L, Liu J, Zhou M (2023). A comprehensive meta-analysis on the association between vitamin C intake and gestational diabetes mellitus: Insights and novel perspectives. Medicine (Baltimore).

[129] Puthanveetil P, Wan A, Rodrigues B (2013). FoxO1 is crucial for sustaining cardiomyocyte metabolism and cell survival. Cardiovasc Res.

[130] Maiese K (2015). Erythropoietin and diabetes mellitus. World J Diabetes.

[131] Zhu B, Cheng H, Li J, Hu Y, Ge X (2025). Decoding mitochondrial stress genes in DCM: towards precision diagnosis and therapy. Hereditas.

[132] Saavedra-Salazar LF, García-Bañuelos J, Monroy-Ramírez HC (2025). Epigenetic regulation of histone 3 acetylation and p53 by SIRT1 overexpression in an experimental model of alveolar epithelium damage. J Biosci.

[133] Feng WJ, Mou J, Liao PP (2024). Alginate oligosaccharides exert protective effects on hydrogen peroxide-induced senescence in H9C2 cardiomyocytes by regulating the redox state of cells. Food Sci Biotechnol.

[134] Golatkar V, Bhatt LK (2024). Artesunate attenuates isoprenaline induced cardiac hypertrophy in rats via SIRT1 inhibiting NF-κB activation. Eur J Pharmacol.

[135] Chauhan W, Zennadi R (2026). Protein arginine methyltransferases in γ-globin regulation and sickle cell disease: emerging connections to oxidative stress. Antioxidants (Basel).

[136] Mill C, Monk BA, Williams H (2014). Wnt5a-induced Wnt1-inducible secreted protein-1 suppresses vascular smooth muscle cell apoptosis induced by oxidative stress. Arterioscler Thromb Vasc Biol.

[137] Munteanu C, Galaction AI, Onose G, Turnea M, Rotariu M (2025). Harnessing gasotransmitters to combat age-related oxidative stress in smooth muscle and endothelial cells. Pharmaceuticals (Basel).

[138] Bjørklund G, Butnariu M, Caunii A, Peana M (2026). Metallothioneins in neurodegenerative diseases: metal homeostasis, autoimmunity, and therapeutic potential. Mol Neurobiol.

[139] Maiese K (2014). Taking aim at Alzheimer’s disease through the mammalian target of rapamycin. Ann Med.

[140] Maiese K (2015). FoxO Transcription Factors and Regenerative Pathways in Diabetes Mellitus. Curr Neurovasc Res.

[141] Yu X, Chen M, Wu J, Song R (2024). Research progress of SIRTs activator resveratrol and its derivatives in autoimmune diseases. Front Immunol.

[142] Okada M, Kim HW, Matsu-ura K, Wang YG, Xu M, Ashraf M (2016). Abrogation of age-induced MicroRNA-195 rejuvenates the senescent mesenchymal stem cells by reactivating telomerase. Stem Cells.

[143] Watroba M, Szukiewicz D (2021). Sirtuins at the service of healthy longevity. Front Physiol.

[144] Gao L, Zheng F, Fu Z, Wang W (2026). Ginsenoside Rb1-enhanced decellularized extracellular matrix hydrogels ameliorates mitochondrial dysfunction and cellular aging in sepsis-induced acute lung injury. J Ginseng Res.

[145] Maiese K (2016). Targeting molecules to medicine with mTOR, autophagy and neurodegenerative disorders. Br J Clin Pharmacol.

[146] Zhao WJ, Liu X, Hu M (2023). Quercetin ameliorates oxidative stress-induced senescence in rat nucleus pulposus-derived mesenchymal stem cells via the miR-34a-5p/SIRT1 axis. World J Stem Cells.

[147] Fauzi A, Wong CZE, Tee PYE (2026). Mechanistic insights into stem cell aging: pathways and processes. Mech Ageing Dev.

[148] Wu X, Zhou Q, Huang Y (2025). Cellular senescence in age-related cardiovascular disease: past and future. Front Aging.

[149] Yamamoto H, Shimomura N, Oura K, Hasegawa Y (2023). Nacre extract from pearl oyster shell prevents D-galactose-induced brain and skin aging. Mar Biotechnol (N Y).

[150] Younis RL, El-Gohary RM, Ghalwash AA (2024). Luteolin mitigates D-galactose-induced brain ageing in rats: SIRT1-mediated neuroprotection. Neurochem Res.

[151] Zhu R, Chen J, Zhang X (2025). Transcriptomics-based analysis of key genes and potential mechanism and therapeutic agents in cadmium-induced esophageal squamous cell carcinoma progression. Sci Rep.

[152] Zhang Z, Liu P, Song Y (2025). Addressing osteoblast senescence: molecular pathways and the frontier of anti-ageing treatments. Clin Transl Med.

[153] Griñán-Ferré C, Servin-Muñoz IV, Palomera-Ávalos V (2024). Changes in gene expression profile with age in SAMP8: identifying transcripts involved in cognitive decline and sporadic Alzheimer’s disease. Genes (Basel).

[154] Jaganathan R, Iyaswamy A, Krishnamoorthi S (2025). Current aspects of targeting cellular senescence for the therapy of neurodegenerative diseases. Front Aging Neurosci.

[155] Jiang T, Du P, Liu D (2025). Exploring the glucose-lowering and anti-inflammatory immune mechanism of artemether by AMPK/mTOR pathway and microbiome based on multi-omics. Front Pharmacol.

[156] Rezvani Kakhki B, Vafadar Moradi E, Vossoughinia S, Yazdanpanah Z, Jalali J, Ghasemzadeh Rahbardar M (2025). Exploring the therapeutic potential of rosemary in metabolic syndrome: from traditional use to modern research. Fitoterapia.

[157] Topiwala A, Nichols TE, Williams LZJ (2023). Telomere length and brain imaging phenotypes in UK Biobank. PLoS One.

[158] Maiese K (2025). Diabetes mellitus and glymphatic dysfunction: roles for oxidative stress, mitochondria, circadian rhythm, artificial intelligence, and imaging. World J Diabetes.

[159] Cardoso S, López IP, Piñeiro-Hermida S, Pichel JG, Moreira PI (2021). IGF1R deficiency modulates brain signaling pathways and disturbs mitochondria and redox homeostasis. Biomedicines.

[160] Kuan XY, Fauzi NSA, Ng KY, Bakhtiar A (2023). Exploring the causal relationship between telomere biology and Alzheimer’s disease. Mol Neurobiol.

[161] Asadinejad H, Taherkhani S, Golboos SM, Azizi Y, Mohammadkhanizadeh A (2025). Targeting neurodegeneration with SGLT2is: from molecular mechanisms to clinical implications. Mol Neurobiol.

[162] Chen YC, Wang WS, Lewis SJG, Wu SL (2024). Fighting against the clock: circadian disruption and Parkinson’s disease. J Mov Disord.

[163] Conze C, Trushina NI, Monteiro-Abreu N (2025). Redox signaling modulates axonal microtubule organization and induces a specific phosphorylation signature of microtubule-regulating proteins. Redox Biol.

[164] Huo H, Zhang A, Shi Y (2025). Kaempferol plays a neuroprotection role by alleviating oxidative stress via AKT/Nrf2/HO-1 pathway and inhibiting apoptosis in intracerebral hemorrhage. Neurochem Res.

[165] Law M, Wang PC, Zhou ZY, Wang Y (2024). From microcirculation to aging-related diseases: a focus on endothelial SIRT1. Pharmaceuticals (Basel).

[166] Maiese K (2023). Cellular metabolism: a fundamental component of degeneration in the nervous system. Biomolecules.

[167] Mirshafa A, Shokrzadeh M, Amiri FT (2025). Tropisetron attenuates D-galactose-induced heart aging in male mice: activation of sirtuin1. Naunyn Schmiedebergs Arch Pharmacol.

[168] Perico L, Remuzzi G, Benigni A (2026). Mitochondria to the rescue: organelle trafficking in renal health and disease. Nephron.

[169] Maiese K, Vincent AM (2000). Membrane asymmetry and DNA degradation: functionally distinct determinants of neuronal programmed cell death. J Neurosci Res.

[170] Williams CJ, Dexter DT (2014). Neuroprotective and symptomatic effects of targeting group III mGlu receptors in neurodegenerative disease. J Neurochem.

[171] Taveira GB, Mello É O, Souza SB (2018). Programmed cell death in yeast by thionin-like peptide from Capsicum annuum fruits involving activation of caspases and extracellular H(+) flux. Biosci Rep.

[172] Almasieh M, Catrinescu MM, Binan L, Costantino S, Levin LA (2017). Axonal degeneration in retinal ganglion cells is associated with a membrane polarity-sensitive redox process. J Neurosci.

[173] Chong ZZ, Kang JQ, Maiese K (2002). Erythropoietin is a novel vascular protectant through activation of Akt1 and mitochondrial modulation of cysteine proteases. Circulation.

[174] Bailey TJ, Fossum SL, Fimbel SM, Montgomery JE, Hyde DR (2010). The inhibitor of phagocytosis, O-phospho-L-serine, suppresses Müller glia proliferation and cone cell regeneration in the light-damaged zebrafish retina. Exp Eye Res.

[175] Shang YC, Chong ZZ, Hou J, Maiese K (2010). Wnt1, FoxO3a, and NF-kappaB oversee microglial integrity and activation during oxidant stress. Cell Signal.

[176] Klionsky DJ, Abdel-Aziz AK, Abdelfatah S (2021). Guidelines for the use and interpretation of assays for monitoring autophagy (4th edition)(1). Autophagy.

[177] Maiese K, Chong ZZ, Shang YC, Wang S (2012). Targeting disease through novel pathways of apoptosis and autophagy. Expert Opin Ther Targets.

[178] Adhikari UK, Khan R, Mikhael M (2023). Therapeutic anti-amyloid β antibodies cause neuronal disturbances. Alzheimers Dement.

[179] Bhattacharya K, Chanu NR, Das D, Kumpakha R, Khanal P, Patil BM, Chikhale R (2026). Neuroinflammation and its impact on autophagy in neurodegeneration. Neuroinflammation and autophagy in neurodegeneration: mechanistic insights into cellular biology and advanced multimodal therapeutics.

[180] Hunjan G, Aran KR (2025). Role of mGluR7 in Alzheimer’s disease: pathophysiological insights and therapeutic approaches. Inflammopharmacology.

[181] Samuels JD, Lukens JR, Price RJ (2024). Emerging roles for ITAM and ITIM receptor signaling in microglial biology and Alzheimer’s disease-related amyloidosis. J Neurochem.

[182] Shang YC, Chong ZZ, Wang S, Maiese K (2012). Prevention of β-amyloid degeneration of microglia by erythropoietin depends on Wnt1, the PI 3-K/mTOR pathway, Bad, and Bcl-xL. Aging (Albany NY).

[183] Lin TK, Huang CR, Lin KJ (2024). Potential roles of hypoxia-inducible factor-1 in Alzheimer’s disease: beneficial or detrimental?. Antioxidants (Basel).

[184] Zhong F, Wu J, Deng Z (2026). ATOX1 overexpression mitigates copper homeostasis in microglia: Implications for Alzheimer’s disease therapy. Genes Dis.

[185] Penning A, Snoeck S, Ormaechea OR (2026). microRNA-132 attenuates inflammation in induced pluripotent stem cell-derived microglia from Alzheimer’s disease patients. Acta Neuropathol Commun.

[186] Liu T, Kong X, Qiao J, Wei J (2025). Decoding Parkinson’s disease: the interplay of cell death pathways, oxidative stress, and therapeutic innovations. Redox Biol.

[187] Tian Y, Ai R, Xiao X, Liu W, Cheng S, Zhu X (2024). Mechanism of the effect of TREM2 on cognitive function in autistic mice. Cell Mol Biol (Noisy-le-grand).

[188] Abdelhafez GF, Boshra SA, Abo-Zalam HB, Radwan SM (2025). Dapagliflozin ameliorates doxorubicin-induced chemobrain and cognitive abnormalities in rats: modulation of AKT/GSK-3β and Wnt/β-catenin pathways. Neurochem Res.

[189] Bian C, Wu Y, Zhang M (2025). Ultraviolet radiation induces caspase cleavage and nuclear translocation of heme oxygenase 1 (HO-1) to activate autophagy in skin keratinocytes. FEBS J.

[190] Maiese K (2016). The bright side of reactive oxygen species: lifespan extension without cellular demise. J Transl Sci.

[191] Elmadhun NY, Sabe AA, Lassaletta AD, Chu LM, Sellke FW (2014). Metformin mitigates apoptosis in ischemic myocardium. J Surg Res.

[192] Meng Y, Ding J, Wang Y (2025). The transcriptional repressor Ctbp2 as a metabolite sensor regulating cardiomyocytes proliferation and heart regeneration. Mol Med.

[193] Cirotti C, Taddei I, Contadini C (2024). NRF2 connects Src tyrosine kinase to ferroptosis resistance in glioblastoma. Life Sci Alliance.

[194] Duarte-Silva E, Meuth SG, Peixoto CA (2023). The role of iron metabolism in the pathogenesis and treatment of multiple sclerosis. Front Immunol.

[195] Gao F, Yang Z, Li J (2025). The miR-34a-5p promotes hippocampal neuronal ferroptosis in epilepsy by regulating SIRT1. Neurochem Res.

[196] Oraby MA, Abdel Mageed SS, Elballal MS (2026). Crocin modulates AKT/mTORC1/ULK1 cascade to attenuate ferritinophagy-induced colonic oxidative injury in ulcerative colitis: network pharmacology, molecular docking, and experimental validation. Phytother Res.

[197] Zheng W, Wen M, Poochali CV, Xie W, Song H (2025). Diagnostic value of the iron apoptosis-related gene in recurrent miscarriage. Medicine (Baltimore).

[198] Zhang M, Liu Q, Meng H (2024). Ischemia-reperfusion injury: molecular mechanisms and therapeutic targets. Signal Transduct Target Ther.

[199] He L, Yang Y, Chen J, Zou P, Li J (2021). Transcriptional activation of ENPP2 by FoxO4 protects cardiomyocytes from doxorubicin-induced toxicity. Mol Med Rep.

[200] Yang L, Cheng CF, Li ZF (2023). Berberine blocks inflammasome activation and alleviates diabetic cardiomyopathy via the miR-18a-3p/Gsdmd pathway. Int J Mol Med.

[201] Yuan Z, Wang F, Wei H (2025). Mechanisms of Talaromyces marneffei induced CNS injury: synergistic roles of tauopathy, pyroptosis, and microglial inflammation. Virulence.

[202] Khedpande N, Barve K (2025). Mitochondrial dysfunction and NLRP3 inflammasome activation in drug-resistant epilepsy: emerging insights and mitochondrial-targeted therapeutic strategies. Naunyn Schmiedebergs Arch Pharmacol.

[203] Ortega AJ, Daniel S, Renwick M (2025). Genetic removal of Nlrp3 protects against age-related and R345W Efemp1-induced basal laminar deposit formation. Cell Death Dis.

[204] Wang Y, Wang Y, Cui Y (2026). FOXO3 upregulates and activates GSDME to trigger myeloma cell pyroptosis. Int J Biol Sci.

[205] Ijaz K, Khan AU, Kamal Y, Irshad N (2024). Effects of dapagliflozin against streptozotocin and isoproterenol-induced heart failure via investigating NLRP3 and PPAR-γ signaling. Pak J Pharm Sci.

[206] Luo L, Li R, Wang G (2022). Age-dependent effects of a high-fat diet combined with dietary advanced glycation end products on cognitive function and protection with voluntary exercise. Food Funct.

[207] Mokhtari B, Badalzadeh R (2021). The potentials of distinct functions of autophagy to be targeted for attenuation of myocardial ischemia/reperfusion injury in preclinical studies: an up-to-date review. J Physiol Biochem.

[208] Wu H, Lan Q, He YX (2025). Programmed cardiomyocyte death in myocardial infarction. Apoptosis.

[209] Mikhel AV, Vasilev DS, Gorbova AV (2025). Effects of prenatal hyperhomocysteinemia on autophagy and mTOR signaling in the developing rat brain. Neurochem J.

[210] Xilouri M, Vogiatzi T, Vekrellis K, Park D, Stefanis L (2009). Abberant alpha-synuclein confers toxicity to neurons in part through inhibition of chaperone-mediated autophagy. PLoS One.

[211] Rapaka D, Bitra VR, Challa SR, Adiukwu PC (2022). mTOR signaling as a molecular target for the alleviation of Alzheimer’s disease pathogenesis. Neurochem Int.

[212] He C, Bassik MC, Moresi V (2012). Exercise-induced BCL2-regulated autophagy is required for muscle glucose homeostasis. Nature.

[213] He C, Zhu H, Li H, Zou MH, Xie Z (2013). Dissociation of Bcl-2-Beclin1 complex by activated AMPK enhances cardiac autophagy and protects against cardiomyocyte apoptosis in diabetes. Diabetes.

[214] Li W, Zhu L, Ruan ZB, Wang MX, Ren Y, Lu W (2019). Nicotinamide protects chronic hypoxic myocardial cells through regulating mTOR pathway and inducing autophagy. Eur Rev Med Pharmacol Sci.

[215] Lu S, Xu D (2013). Cold stress accentuates pressure overload-induced cardiac hypertrophy and contractile dysfunction: role of TRPV1/AMPK-mediated autophagy. Biochem Biophys Res Commun.

[216] Liu J, Zhang Y, Tang J (2025). Exendin-4 alleviates diabetic retinopathy by activating autophagy via regulation of the adenosine monophosphate-activated protein kinase/sirtuin 1 pathway. J Mol Histol.

[217] Pan Y, Yang XH, Guo LL, Gu YH, Qiao QY, Jin HM (2017). Erythropoietin reduces insulin resistance via regulation of its receptor-mediated signaling pathways in db/db mice skeletal muscle. Int J Biol Sci.

[218] Maiese K (2020). Cognitive impairment with diabetes mellitus and metabolic disease: innovative insights with the mechanistic target of rapamycin and circadian clock gene pathways. Expert Rev Clin Pharmacol.

[219] Maiese K, Li F, Chong ZZ (2005). New avenues of exploration for erythropoietin. JAMA.

[220] Zhao L, Han Y, Wang Y, Ke T, Huang S (2025). Ischemic postconditioning ameliorates diabetic cerebral ischemia via activating the brain-derived neurotrophic factor-tropomyosin receptor kinase B-hypoxia-inducible factor 1α-Bcl-2/adenovirus E1B 19-kDa-interacting protein 3 pathway to induce microglial mitophagy and suppress A1 astrocyte-mediated neuroinflammation. Front Endocrinol (Lausanne).

[221] Gong Q, Wang H, Yu P, Qian T, Xu X (2021). Protective or harmful: the dual roles of autophagy in diabetic retinopathy. Front Med (Lausanne).

[222] Bargiela A, Cerro-Herreros E, Fernandez-Costa JM, Vilchez JJ, Llamusi B, Artero R (2015). Increased autophagy and apoptosis contribute to muscle atrophy in a myotonic dystrophy type 1 Drosophila model. Dis Model Mech.

[223] Barthels D, Prateeksha P, Nozohouri S (2023). Dental pulp-derived stem cells preserve astrocyte health during induced gliosis by modulating mitochondrial activity and functions. Cell Mol Neurobiol.

[224] Chiu YC, Hsieh TJ, Ma CH, Jou IM, Wu CH (2025). Oxidative stress-associated autophagy correlates to the disease severity of de Quervain’s disease. Eur J Rheumatol.

[225] Di T, Zhou Z, Liu F, Chen Y, Wang L (2024). Autophagy and circadian rhythms: interactions and clinical implications. Biocell.

[226] Maiese K (2017). Moving to the rhythm with clock (circadian) genes, autophagy, mTOR, and SIRT1 in degenerative disease and cancer. Curr Neurovasc Res.

[227] Maiese K (2021). Neurodegeneration, memory loss, and dementia: the impact of biological clocks and circadian rhythm. Front Biosci (Landmark Ed).

[228] Dong Y, Chen H, Gao J, Liu Y, Li J, Wang J (2019). Molecular machinery and interplay of apoptosis and autophagy in coronary heart disease. J Mol Cell Cardiol.

[229] Wagner C, Tillack D, Simonis G, Strasser RH, Weinbrenner C (2010). Ischemic post-conditioning reduces infarct size of the in vivo rat heart: role of PI3-K, mTOR, GSK-3beta, and apoptosis. Mol Cell Biochem.

[230] Peng N, Meng N, Wang S (2014). An activator of mTOR inhibits oxLDL-induced autophagy and apoptosis in vascular endothelial cells and restricts atherosclerosis in apolipoprotein E^−^/^−^ mice. Sci Rep.

[231] Bennis Y, Sarlon-Bartoli G, Guillet B (2012). Priming of late endothelial progenitor cells with erythropoietin before transplantation requires the CD131 receptor subunit and enhances their angiogenic potential. J Thromb Haemost.

[232] Busch S, Kannt A, Kolibabka M (2014). Systemic treatment with erythropoietin protects the neurovascular unit in a rat model of retinal neurodegeneration. PLoS One.

[233] Hou J, Wang S, Shang YC, Chong ZZ, Maiese K (2011). Erythropoietin employs cell longevity pathways of SIRT1 to foster endothelial vascular integrity during oxidant stress. Curr Neurovasc Res.

[234] Yu DF, Zhu LH, Jiang L (2017). Recombinant human erythropoietin augments neovascularization responses in a neonatal rat model of premature brain damage by phosphatidylinositol 3 kinase/Akt pathway. Chin Med J (Engl).

[235] Ferrara-Romeo I, Martinez P, Saraswati S (2020). The mTOR pathway is necessary for survival of mice with short telomeres. Nat Commun.

[236] Maiese K (2001). The dynamics of cellular injury: transformation into neuronal and vascular protection. Histol Histopathol.

[237] Ong WY, Wu YJ, Farooqui T, Farooqui AA (2018). Qi Fu Yin-a Ming dynasty prescription for the treatment of dementia. Mol Neurobiol.

[238] Guo S, Zhou Y, Xing C (2012). The vasculome of the mouse brain. PLoS One.

[239] Bagri K, Kumari S, Bishnoi M, Deshmukh R, Malik J, Mandal SC, Sarup P, Lin GD, Li RW (2026). Molecular basis for the protective effect of dietary components against Alzheimer’s disease. Diet and Alzheimer’s disease: let food be our medicine.

[240] Filley CM, Rollins YD, Anderson CA (2007). The genetics of very early onset Alzheimer disease. Cogn Behav Neurol.

[241] Fessel J (2024). Personalized, precision medicine to cure Alzheimer’s dementia: approach #1. Int J Mol Sci.

[242] Lalmohamed A, Bazelier MT, Van Staa TP (2012). Causes of death in patients with multiple sclerosis and matched referent subjects: a population-based cohort study. Eur J Neurol.

[243] Staff NP, Lucchinetti CF, Keegan BM (2009). Multiple sclerosis with predominant, severe cognitive impairment. Arch Neurol.

[244] Yap SM, Davenport L, Cogley C (2023). Word finding, prosody and social cognition in multiple sclerosis. J Neuropsychol.

[245] Gheibi S, YahyaZadeh M, Karimipour M, Ghazavi A, Kashfi K (2025). High-fat diet exacerbates neuroinflammation and disease severity in a rat model of multiple sclerosis. Int J Prev Med.

[246] Naserpour L, Jalise SZ, Khoshandam M, Hosseinzadeh F (2025). Molecular mechanism and the main signaling pathways in the development and progression of multiple sclerosis. Exp Cell Res.

[247] Roberts RO, Knopman DS, Geda YE, Cha RH, Roger VL, Petersen RC (2010). Coronary heart disease is associated with non-amnestic mild cognitive impairment. Neurobiol Aging.

[248] Ahmed F, Awan AI, Kamel O, Waheed N, Afzaal Z, Aman K (2026). Depression and cardiovascular burden in aging populations: insights from the national health and nutrition examination survey 2010-2020 on symptom frequency, sociodemographic disparities, and modifiable risk factors. Prim Care Companion CNS Disord.

[249] Osuna AR, Kennedy J, Zhou C, Christakis DA (2026). Anxiety, depression, and care barriers in adults with intellectual and developmental disabilities. JAMA Netw Open.

[250] Dos Santos Silva J, La Rosa F, Dereskewicz E (2026). Neuroanatomical correlates of depressive symptoms in early multiple sclerosis. Neurol Open Access.

[251] Amanollahi M, Jameie M, Heidari A, Rezaei N (2023). The dialogue between neuroinflammation and adult neurogenesis: mechanisms involved and alterations in neurological diseases. Mol Neurobiol.

[252] Na D, Lim DH, Hong JS (2023). A multi-layered network model identifies Akt1 as a common modulator of neurodegeneration. Mol Syst Biol.

[253] Casoli T, Di Stefano G, Giorgetti B (2007). Release of beta-amyloid from high-density platelets: implications for Alzheimer’s disease pathology. Ann N Y Acad Sci.

[254] Cardoso SM, Proença MT, Santos S, Santana I, Oliveira CR (2004). Cytochrome c oxidase is decreased in Alzheimer’s disease platelets. Neurobiol Aging.

[255] Kruman, II, Culmsee C, Chan SL (2000). Homocysteine elicits a DNA damage response in neurons that promotes apoptosis and hypersensitivity to excitotoxicity. J Neurosci.

[256] Kell DB, Pretorius E (2018). No effects without causes: the iron dysregulation and dormant microbes hypothesis for chronic, inflammatory diseases. Biol Rev Camb Philos Soc.

[257] Ojo JO, Reed JM, Crynen G (2021). APOE genotype dependent molecular abnormalities in the cerebrovasculature of Alzheimer’s disease and age-matched non-demented brains. Mol Brain.

[258] Maiese K (2020). Nicotinamide: oversight of metabolic dysfunction through SIRT1, mTOR, and clock genes. Curr Neurovasc Res.

[259] Toniolo S, Scarioni M, Di Lorenzo F (2021). Dementia and COVID-19, a bidirectional liaison: risk factors, biomarkers, and optimal health care. J Alzheimers Dis.

[260] Zhou Y, Xu J, Hou Y (2021). Network medicine links SARS-CoV-2/COVID-19 infection to brain microvascular injury and neuroinflammation in dementia-like cognitive impairment. Alzheimers Res Ther.

[261] Abreu MM, Spitz VH, Smadja DM (2026). Diabetes, protein misfolding, and heat stress: molecular insights and translational perspectives. TH Open.

[262] Karamzad N, Maleki V, Carson-Chahhoud K, Azizi S, Sahebkar A, Gargari BP (2020). A systematic review on the mechanisms of vitamin K effects on the complications of diabetes and pre-diabetes. Biofactors.

[263] Meneses do Rêgo AC, Araújo-Filho I (2024). Post-acute COVID-19 syndrome and stroke: post-acute COVID-19 syndrome and stroke. J Surg Clin Res.

[264] Aravapally PSN, Chandrasekar N, Verma A, Shah RP (2025). Strategic approaches to assess and quantify the oxidative stress biomarkers in complex biological systems. Bioanalysis.

[265] Zhang Y, Yao L, Lyu Y, Tang Z, Liu X, Duan X (2025). From bench to bedside: therapeutic potential of natural antioxidants in diabetic neuropathy. Front Cell Dev Biol.

[266] Leenders F, de Koning EJP, Carlotti F (2024). Pancreatic β-cell identity change through the lens of single-cell omics research. Int J Mol Sci.

[267] Liu S, Liu T, Li J, Hong J, Moosavi-Movahedi AA, Wei J (2025). Type 2 diabetes mellitus exacerbates pathological processes of Parkinson’s disease: insights from signaling pathways mediated by insulin receptors. Neurosci Bull.

[268] Maiese K (2015). New insights for oxidative stress and diabetes mellitus. Oxid Med Cell Longev.

[269] Maiese K (2020). Prospects and perspectives for WISP1 (CCN4) in diabetes mellitus. Curr Neurovasc Res.

[270] Moheghi A, Bagheri Tadi F, Noori Mougehi SM (2026). Targeting oxidative stress in diabetic wound repair: combined photobiomodulation and adipose-derived stem cell therapy. Eur J Plast Surg.

[271] Caiazzo E, Delre P, Cerchia C (2026). Structural and functional characterization of chemically modified antisense oligonucleotides targeting miR-214-3p in endothelial cells. Bioorg Chem.

[272] Wong JH, Lai KO, Barron AM (2026). Unlocking therapies for neurodegeneration: targeting mitochondrial dysfunction and oxidative stress in Alzheimer’s disease. Curr Opin Physiol.

[273] Liu C, Niu X, Xu Y, Liu W (2026). Multidimensional regulatory mechanisms and targeted therapeutic strategies for inhibited keratinocyte proliferation in diabetic wounds. Drug Des Devel Ther.

[274] Maiorana A, O’Driscoll G, Goodman C, Taylor R, Green D (2002). Combined aerobic and resistance exercise improves glycemic control and fitness in type 2 diabetes. Diabetes Res Clin Pract.

[275] Jang W, Park J, Shin KJ (2014). Safety and efficacy of recombinant human erythropoietin treatment of non-motor symptoms in Parkinson’s disease. J Neurol Sci.

[276] Wright JW, Kawas LH, Harding JW (2015). The development of small molecule angiotensin IV analogs to treat Alzheimer’s and Parkinson’s diseases. Prog Neurobiol.

[277] Martinez-Nunez AE, Mills KA, Seemiller J, Morrow CB, Pontone GM, Mahajan A (2026). Early autonomic burden in prodromal Parkinson’s disease predicts cognitive impairment. Mov Disord.

[278] Norman JE, Nuthikattu S, Milenkovic D, Villablanca AC (2025). Cell- and sex-specificity in the transcriptomic response of the hippocampal neurovascular unit to obesity. Commun Biol.

[279] Wei S, Cheng L, Shen C (2025). Necroptosis in vascular cognitive impairment: mechanisms and therapeutic potential. Front Aging Neurosci.

[280] Barlattani T, Cavatassi A, Bologna A (2025). Glymphatic system and psychiatric disorders: need for a new paradigm?. Front Psychiatry.

[281] Finn Q, Pascual B, Schulz PE, Masdeu JC (2026). β-Amyloid PET signal reduction in prior ARIA-E regions after antiamyloid therapy for Alzheimer disease. AJNR Am J Neuroradiol.

[282] Park KM, Kim KT, Lee DA, Motamedi GK, Cho YW (2023). Glymphatic system dysfunction in restless legs syndrome: evidenced by diffusion tensor imaging along the perivascular space. Sleep.

[283] Theoharides TC (2022). Could SARS-CoV-2 spike protein be responsible for long-COVID syndrome?. Mol Neurobiol.

[284] Lim RK, Wambier CG, Goren A (2020). Are night shift workers at an increased risk for COVID-19?. Med Hypotheses.

[285] Cardinali DP, Brown GM, Reiter RJ, Pandi-Perumal SR (2020). Elderly as a high-risk group during COVID-19 pandemic: effect of circadian misalignment, sleep dysregulation and melatonin administration. Sleep Vigil.

[286] Gu S, Li Y, Jiang Y, Huang JH, Wang F (2022). Glymphatic dysfunction induced oxidative stress and neuro-inflammation in major depression disorders. Antioxidants (Basel).

[287] Maiese K (2021). Cognitive impairment and dementia: gaining insight through circadian clock gene pathways. Biomolecules.

[288] Tian B, Zhao C, Liang JL (2024). Glymphatic function and its influencing factors in different glucose metabolism states. World J Diabetes.

[289] Abdalla MMI (2024). Insulin resistance as the molecular link between diabetes and Alzheimer’s disease. World J Diabetes.

[290] Burillo J, Marqués P, Jiménez B, González-Blanco C, Benito M, Guillén C (2021). Insulin resistance and diabetes mellitus in Alzheimer’s disease. Cells.

[291] Ancu O, Hauge-Evans AC, Draicchio F (2026). High protein ingestion does not affect whole-body insulin sensitivity in individuals with overweight or obesity. J Endocr Soc.

[292] Maiese K (2016). Novel nervous and multi-system regenerative therapeutic strategies for diabetes mellitus with mTOR. Neural Regen Res.

[293] Ciardullo S, Muraca E, Bianconi E (2023). Diabetes mellitus is associated with higher serum neurofilament light chain levels in the general US population. J Clin Endocrinol Metab.

[294] Carobene A, Maiese K, Abou-Diwan C (2023). Biological variation estimates for serum neurofilament light chain in healthy subjects. Clin Chim Acta.

[295] Gut N, Piecha G, Aldebssi F (2013). Erythropoietin combined with ACE inhibitor prevents heart remodeling in 5/6 nephrectomized rats independently of blood pressure and kidney function. Am J Nephrol.

[296] Hefer D, Yi T, Selby DE (2012). Erythropoietin induces positive inotropic and lusitropic effects in murine and human myocardium. J Mol Cell Cardiol.

[297] Jun JH, Shin EJ, Kim JH, Kim SO, Shim JK, Kwak YL (2013). Erythropoietin prevents hypoxia-induced GATA-4 ubiquitination via phosphorylation of serine 105 of GATA-4. Biol Pharm Bull.

[298] Lu MJ, Chen YS, Huang HS, Ma MC (2012). Erythropoietin alleviates post-ischemic injury of rat hearts by attenuating nitrosative stress. Life Sci.

[299] Yuksel IO, Cagirci G, Koklu E (2016). Erythropoietin stimulates the coronary collateral development in patients with coronary chronic total occlusion. Neth Heart J.

[300] Çalışkan H, Önal D, Nalçacı E (2024). Darbepoetin alpha has an anxiolytic and anti-neuroinflammatory effect in male rats. BMC Immunol.

[301] Vinberg M, Højman P, Pedersen BK, Kessing LV, Miskowiak KW (2018). Effects of erythropoietin on body composition and fat-glucose metabolism in patients with affective disorders. Acta Neuropsychiatr.

[302] Fessel J (2023). Cure of Alzheimer’s dementia requires addressing all of the affected brain cell types. J Clin Med.

[303] Garrigue P, Hache G, Bennis Y (2016). Erythropoietin pretreatment of transplanted endothelial colony-forming cells enhances recovery in a cerebral ischemia model by increasing their homing ability: a SPECT/CT study. J Nucl Med.

[304] Wagenaar N, de Theije CGM, de Vries LS, Groenendaal F, Benders M, Nijboer CHA (2018). Promoting neuroregeneration after perinatal arterial ischemic stroke: neurotrophic factors and mesenchymal stem cells. Pediatr Res.

[305] Yang K, Zhang L, Chen W (2023). Expression of EPO and related factors in the liver and kidney of plain and Tibetan sheep. Histol Histopathol.

[306] Shiratani Y, Furuya T, Nagashima Y (2026). Effects of decompression surgery and erythropoietin combination on a rat model of compressive myelopathy. J Orthop Sci.

[307] Lee HJ, Koh SH, Song KM, Seol IJ, Park HK (2016). The Akt/mTOR/p70S6K pathway is involved in the neuroprotective effect of erythropoietin on hypoxic/ischemic brain injury in a neonatal rat model. Neonatology.

[308] Moteki H, Ogihara M, Kimura M (2026). Intracellular signaling pathways for erythropoietin-induced cell proliferation in primary cultured hepatocytes. Biol Pharm Bull.

[309] Senousy MA, Hanafy ME, Shehata N, Rizk SM (2022). Erythropoietin and bacillus calmette-guérin vaccination mitigate 3-nitropropionic acid-induced Huntington-like disease in rats by modulating the PI3K/Akt/mTOR/P70S6K pathway and enhancing the autophagy. ACS Chem Neurosci.

[310] Grimaldi L, Bovi E, Formisano R, Sancesario G (2024). ApoE: the non-protagonist actor in neurological diseases. Genes (Basel).

[311] Hou SJ, Zhang SX, Li Y, Xu SY (2023). Rapamycin responds to Alzheimer’s disease: a potential translational therapy. Clin Interv Aging.

[312] Mishra P, Davies DA, Albensi BC (2023). The interaction between NF-κB and estrogen in Alzheimer’s disease. Mol Neurobiol.

[313] Tang H, Shaaban CE, DeKosky ST (2024). Association of education attainment, smoking status, and alcohol use disorder with dementia risk in older adults: a longitudinal observational study. Alzheimers Res Ther.

[314] Xu M, Zhao J, Zhang Y (2016). Apolipoprotein E gene variants and risk of coronary heart disease: a meta-analysis. Biomed Res Int.

[315] Chung YE, Chung RH, Hsu CC (2026). APOE ε4 and accelerated cognitive decline among cognitively healthy middle-aged and older adults. JAMA Netw Open.

[316] Naseri A, Baghernezhad K, Seyedi-Sahebari S (2022). The association of apolipoprotein E (ApoE) genotype and cognitive outcomes in multiple sclerosis; a systematic review and meta-analysis. Mult Scler Relat Disord.

[317] Momkute L, Vilkeviciute A, Gedvilaite G, Dubinskaite G, Kriauciuniene L, Liutkeviciene R (2022). Association of APOE serum levels and APOE ε2, ε3, and ε4 alleles with optic neuritis. Genes (Basel).

[318] Kurki SN, Kantonen J, Kaivola K (2021). APOE ε4 associates with increased risk of severe COVID-19, cerebral microhaemorrhages and post-COVID mental fatigue: a Finnish biobank, autopsy and clinical study. Acta Neuropathol Commun.

[319] Safdari Lord J, Soltani Rezaiezadeh J, Yekaninejad MS, Izadi P (2022). The association of APOE genotype with COVID-19 disease severity. Sci Rep.

[320] Lee G, Pollard HB, Arispe N (2002). Annexin 5 and apolipoprotein E2 protect against Alzheimer’s amyloid-beta-peptide cytotoxicity by competitive inhibition at a common phosphatidylserine interaction site. Peptides.

[321] de la Monte SM, Tong M, Carlson RI, Sutherland G (2026). Impaired brain incretin and gut hormone expression in human alcohol-related brain damage: opportunities for therapeutic targeting. Biomolecules.

[322] Li L (2017). The molecular mechanism of glucagon-like peptide-1 therapy in Alzheimer’s disease, based on a mechanistic target of rapamycin pathway. CNS Drugs.

[323] Barakat GM, Moustafa ME, Bikhazi AB (2012). Effects of selenium and exendin-4 on glucagon-like peptide-1 receptor, IRS-1, and Raf-1 in the liver of diabetic rats. Biochem Genet.

[324] Chen Z, Zhong C (2013). Decoding Alzheimer’s disease from perturbed cerebral glucose metabolism: implications for diagnostic and therapeutic strategies. Prog Neurobiol.

[325] Palleria C, Leporini C, Maida F (2016). Potential effects of current drug therapies on cognitive impairment in patients with type 2 diabetes. Front Neuroendocrinol.

[326] Wang P, Yan Z, Zhong J (2012). Transient receptor potential vanilloid 1 activation enhances gut glucagon-like peptide-1 secretion and improves glucose homeostasis. Diabetes.

[327] Kimura R, Okouchi M, Kato T (2013). Epidermal growth factor receptor transactivation is necessary for glucagon-like peptide-1 to protect PC12 cells from apoptosis. Neuroendocrinology.

[328] Miao XY, Gu ZY, Liu P (2013). The human glucagon-like peptide-1 analogue liraglutide regulates pancreatic beta-cell proliferation and apoptosis via an AMPK/mTOR/P70S6K signaling pathway. Peptides.

[329] Tomas E, Habener JF (2010). Insulin-like actions of glucagon-like peptide-1: a dual receptor hypothesis. Trends Endocrinol Metab.

[330] Chen Z, Yao K, Wang X (2025). Acupuncture promotes muscle cells ATP metabolism in ST36 acupoint local exerting effect by activating TRPV1/CaMKII/AMPK/PGC1α signaling pathway. Chin Med.

[331] Fan Y, Wang P, Jiang C, Chen J, Zhao M, Liu J (2024). Tet1-mediated activation of the Ampk signaling by Trpv1 DNA hydroxymethylation exerts neuroprotective effects in a rat model of Parkinson’s disease. Funct Integr Genomics.

[332] Maiese K (2017). Warming up to new possibilities with the capsaicin receptor TRPV1: mTOR, AMPK, and erythropoietin. Curr Neurovasc Res.

[333] Santos GX, Dos Anjos-Garcia T, Vieira ACJ, Galdino G (2025). Spinal involvement of TRPV1 and PI3K/AKT/mTOR pathway during chronic postoperative pain in mice. Brain Sci.

[334] Chi KY, Chang Y, Song J, Chen CM, Perng PS, Lin HM (2026). GLP-1 receptor agonist use and survival among patients with type 2 diabetes and brain metastases. JAMA Netw Open.

[335] Zhou J, Wu J, Zheng F, Jin M, Li H (2015). Glucagon-like peptide-1 analog-mediated protection against cholesterol-induced apoptosis via mammalian target of rapamycin activation in pancreatic βTC-6 cells -1mTORβTC-6. J Diabetes.

[336] Magdy YM, Kamar SA, Habib MZ, Rady HY, Rabei MR, Khedr S (2025). Liraglutide improves depressive and cognitive deficits in a high-fat diet rat model of obesity: the role of hippocampal autophagy and the PI3K/Akt/mTOR pathway. Psychopharmacology (Berl).

[337] Sayed NH, Fathy N, Kortam MA, Rabie MA, Mohamed AF, Kamel AS (2020). Vildagliptin attenuates Huntington’s disease through activation of GLP-1 receptor/PI3K/Akt/BDNF pathway in 3-nitropropionic acid rat model. Neurotherapeutics.

[338] Huang J, Chen Z, Wu Z (2023). Geniposide stimulates autophagy by activating the GLP-1R/AMPK/mTOR signaling in osteoarthritis chondrocytes. Biomed Pharmacother.

[339] Schweitzer K (2026). What to know about the WHO’s new GLP-1 drug guideline. JAMA.

[340] Ho MJ, Liew CF, Tan NS, Morita A, Oon HH (2026). Glucagon-like peptide-1 receptor agonists (GLP-1RAs) in dermatology: cutaneous adverse events and emerging efficacy in inflammatory skin diseases. Expert Rev Clin Immunol.

[341] Aboukaoud M, Hoch B, Weiser M, Amiaz R (2026). Depressed mood and suicidal thoughts reporting with GLP-1 receptor agonists in type 2 diabetes: A WHO VigiBase study. J Affect Disord.

[342] Maiese K (2026). Osteoarthritis and dementia: contrasting disorders driven by mutual pathways of autophagy, mTOR, GLP-1, AMPK, Wnt, and WISP1. Expert Rev Clin Pharmacol.

[343] Apiraksattayakul S, Pingaew R, Prachayasittikul V (2024). Neuroprotective potential of aminonaphthoquinone derivatives against amyloid beta-induced neuronal cell death through modulation of SIRT1 and BACE1. Neurochem Res.

[344] Ding MR, Qu YJ, Hu B, An HM (2022). Signal pathways in the treatment of Alzheimer’s disease with traditional Chinese medicine. Biomed Pharmacother.

[345] Jahan R, Yousaf M, Khan H (2023). Zinc ortho methyl carbonodithioate improved pre and post-synapse memory impairment via SIRT1/p-JNK pathway against scopolamine in adult mice. J Neuroimmune Pharmacol.

[346] Jongwachirachai P, Ruankham W, Apiraksattayakul S (2024). Neuroprotective properties of coriander-derived compounds on neuronal cell damage under oxidative stress-induced SH-SY5Y neuroblastoma and in silico ADMET analysis. Neurochem Res.

[347] Matysek A, Sun L, Kimmantudawage SP, Feng L, Maier AB (2023). Targeting impaired nutrient sensing via the sirtuin pathway with novel compounds to prevent or treat dementia: a systematic review. Ageing Res Rev.

[348] Penteado AB, Hassanie H, Gomes RA, Silva Emery FD, Goulart Trossini GH (2023). Human sirtuin 2 inhibitors, their mechanisms and binding modes. Future Med Chem.

[349] Dhillon VS, Shahid M, Deo P, Fenech M (2024). Reduced SIRT1 and SIRT3 and lower antioxidant capacity of seminal plasma is associated with shorter sperm telomere length in oligospermic men. Int J Mol Sci.

[350] Han X, Peng M, Wang X (2025). Role of the AMPK/PGC-1α/SIRT3-mediated mitochondrial dysfunction in the neurotoxicity of methanol. Mol Neurobiol.

[351] Khan I, Kaur S, Rishi AK, Boire B, Aare M, Singh M (2024). Cannabidiol and beta-caryophyllene combination attenuates diabetic neuropathy by inhibiting NLRP3 inflammasome/NFκB through the AMPK/sirT3/Nrf2 axis. Biomedicines.

[352] Chen YR, Fang SR, Fu YC, Zhou XH, Xu MY, Xu WC (2010). Calorie restriction on insulin resistance and expression of SIRT1 and SIRT4 in rats. Biochem Cell Biol.

[353] Mori M, Cazzaniga G, Meneghetti F, Villa S, Gelain A (2022). Insights on the modulation of SIRT5 activity: a challenging balance. Molecules.

[354] Wasserfurth P, Nebl J, Rühling MR (2021). Impact of dietary modifications on plasma sirtuins 1, 3 and 5 in older overweight individuals undergoing 12-weeks of circuit training. Nutrients.

[355] Wang Z, Wu Q, Wang H (2022). Diosgenin protects against podocyte injury in early phase of diabetic nephropathy through regulating SIRT6. Phytomedicine.

[356] Zhang GZ, Deng YJ, Xie QQ (2020). Sirtuins and intervertebral disc degeneration: Roles in inflammation, oxidative stress, and mitochondrial function. Clin Chim Acta.

[357] Li X, Luo X, Cao X (2025). Sirtuins in Parkinson’s disease: Molecular mechanisms and pathophysiological roles. Ageing Res Rev.

[358] Maiese K (2018). The mechanistic target of rapamycin (mTOR) and the silent mating-type information regulation 2 homolog 1 (SIRT1): oversight for neurodegenerative disorders. Biochem Soc Trans.

[359] Maiese K, Maiese K (2021). Chapter 1 - Sirtuins in metabolic disease: innovative therapeutic strategies with SIRT1, AMPK, mTOR, and nicotinamide. Sirtuin biology in cancer and metabolic disease.

[360] Chen Y, Han SY, Zhong ZX (2026). Coordinating metabolic reprogramming and exosome secretion: A dual role for SIRT1/HIF-1α under hypoxia in golden pompano. Aquaculture.

[361] Wang Y, Cheng C, Li L (2026). A novel gentiopicroside derivative clears phosphorylation tau to improve memory of AD mice by targeting AMPK and increase of gut neuroprotective metabolites. Phytomedicine.

[362] Zhou D, Yu G, Fu X (2026). SIRT1 regulates dermal fibroblast senescence via impaired deacetylase function and mitochondrial dysfunction during skin aging induced by chronic oral cadmium exposure. Front Public Health.

[363] Jaiswal S, Mishra V, Majumder S, Wangikar PP, Sengupta S (2025). Metabolomic profiling reveals grade-specific niacinamide accumulation and its therapeutic potential via SIRT1-CD38-EMT axis modulation in cervical cancer progression. Biochim Biophys Acta Mol Cell Res.

[364] Chen M, Zhang H, Ji P (2025). Therapeutic potential of ACMSD inhibitors in NAD+ deficient diseases. Drugs Drug Candidates.

[365] Ministrini S, Puspitasari YM, Beer G, Liberale L, Montecucco F, Camici GG (2021). Sirtuin 1 in endothelial dysfunction and cardiovascular aging. Front Physiol.

[366] Sun WD, Zhu XJ, Li JJ, Mei YZ, Li WS, Li JH (2024). Nicotinamide N-methyltransferase (NNMT): a novel therapeutic target for metabolic syndrome. Front Pharmacol.

[367] Fangma Y, Wan H, Shao C, Jin L, He Y (2023). Research progress on the role of sirtuin 1 in cerebral ischemia. Cell Mol Neurobiol.

[368] Chong MC, Silva A, James PF, Wu SSX, Howitt J (2022). Exercise increases the release of NAMPT in extracellular vesicles and alters NAD(+) activity in recipient cells. Aging Cell.

[369] Babighian S, Gattazzo I, Zanella MS (2024). Nicotinamide: bright potential in glaucoma management. Biomedicines.

[370] Chong ZZ, Lin SH, Maiese K (2004). The NAD+ precursor nicotinamide governs neuronal survival during oxidative stress through protein kinase B coupled to FOXO3a and mitochondrial membrane potential. J Cereb Blood Flow Metab.

[371] D’Angelo A, Vitiello L, Lixi F (2024). Optic nerve neuroprotection in glaucoma: a narrative review. J Clin Med.

[372] Du X, Cui Z, Zhang R (2023). The effects of rumen-protected choline and rumen-protected nicotinamide on liver transcriptomics in periparturient dairy cows. Metabolites.

[373] Maiese K (2021). Nicotinamide as a foundation for treating neurodegenerative disease and metabolic disorders. Curr Neurovasc Res.

[374] Ramírez-Cruz A, Gómez-González B, Baiza-Gutman LA (2023). Nicotinamide, an acetylcholinesterase uncompetitive inhibitor, protects the blood–brain barrier and improves cognitive function in rats fed a hypercaloric diet. Eur J Pharmacol.

[375] Jalgaonkar MP, Parmar UM, Kulkarni YA, Oza MJ (2022). SIRT1-FOXOs activity regulates diabetic complications. Pharmacol Res.

[376] Avalos JL, Bever KM, Wolberger C (2005). Mechanism of sirtuin inhibition by nicotinamide: altering the NAD(+) cosubstrate specificity of a Sir2 enzyme. Mol Cell.

[377] Fulco M, Cen Y, Zhao P (2008). Glucose restriction inhibits skeletal myoblast differentiation by activating SIRT1 through AMPK-mediated regulation of Nampt. Dev Cell.

[378] Jackson MD, Schmidt MT, Oppenheimer NJ, Denu JM (2003). Mechanism of nicotinamide inhibition and transglycosidation by Sir2 histone/protein deacetylases. J Biol Chem.

[379] Jobst M, Kiss E, Gerner C, Marko D, Del Favero G (2023). Activation of autophagy triggers mitochondrial loss and changes acetylation profile relevant for mechanotransduction in bladder cancer cells. Arch Toxicol.

[380] Zhou R, Barnes K, Gibson S, Fillmore N (2024). Dual-edged role of SIRT1 in energy metabolism and cardiovascular disease. Am J Physiol Heart Circ Physiol.

[381] Kostić M, Korićanac G, Tepavčević S (2023). Low-intensity exercise affects cardiac fatty acid oxidation by increasing the nuclear content of PPARα, FOXO1, and Lipin1 in fructose-fed rats. Metab Syndr Relat Disord.

[382] Liu X, Chen H, Zhu W (2014). Transplantation of SIRT1-engineered aged mesenchymal stem cells improves cardiac function in a rat myocardial infarction model. J Heart Lung Transplant.

[383] Charles S, Raj V, Arokiaraj J, Mala K (2017). Caveolin1/protein arginine methyltransferase1/sirtuin1 axis as a potential target against endothelial dysfunction. Pharmacol Res.

[384] Piao S, Lee I, Jin SA (2021). SIRT1 activation attenuates the cardiac dysfunction induced by endothelial cell-specific deletion of CRIF1. Biomedicines.

[385] Trujillo-Rangel W, Acuña-Vaca S, Padilla-Ponce DJ (2024). Modulation of the circadian rhythm and oxidative stress as molecular targets to improve vascular dementia: a pharmacological perspective. Int J Mol Sci.

[386] Maiese K, Chong ZZ, Shang YC, Wang S (2013). Novel directions for diabetes mellitus drug discovery. Expert Opin Drug Discov.

[387] Tabibzadeh S (2021). Signaling pathways and effectors of aging. Front Biosci (Landmark Ed).

[388] Ye M, Zhao Y, Wang Y (2022). NAD(H)-loaded nanoparticles for efficient sepsis therapy via modulating immune and vascular homeostasis. Nat Nanotechnol.

[389] Cui L, Guo J, Zhang Q (2017). Erythropoietin activates SIRT1 to protect human cardiomyocytes against doxorubicin-induced mitochondrial dysfunction and toxicity. Toxicol Lett.

[390] Wu H, Wang H, Zhang W (2015). rhEPO affects apoptosis in hippocampus of aging rats by upregulating SIRT1. Int J Clin Exp Pathol.

[391] Ahmed R, Safa MR, Zahid ZI (2025). Association of SIRT1 rs3758391 polymorphism with T2DM in bangladeshi population: evidence from a case-control study and meta-analysis. Health Sci Rep.

[392] Anggreini P, Kuncoro H, Sumiwi SA, Levita J (2023). Role of the AMPK/SIRT1 pathway in non-alcoholic fatty liver disease (Review). Mol Med Rep.

[393] Letsiou S, Prountzou E, Vougiouklaki D (2025). Association of SIRT1 promoter polymorphisms with type 2 diabetes mellitus and pregnancy-related complications in the Greek population. Genes (Basel).

[394] Khazaei AH, Bozorgi A, Ghanbari E, Bozorgi M, Khazaei M (2025). Trifolium pratense-derived exosome improved serum biochemical parameters and pancreatic genes in STZ-induced diabetic rats. Endocrinol Diabetes Metab.

[395] Du LL, Chai DM, Zhao LN (2015). AMPK activation ameliorates Alzheimer’s disease-like pathology and spatial memory impairment in a streptozotocin-induced Alzheimer’s disease model in rats. J Alzheimers Dis.

[396] Maiese K (2016). Forkhead transcription factors: new considerations for Alzheimer’s disease and dementia. J Transl Sci.

[397] Qian S, Gu J, Dai W (2018). Sirt1 enhances tau exon 10 inclusion and improves spatial memory of Htau mice. Aging (Albany NY).

[398] Esmaeili M, Nasr-Esfahani MH, Shoaraye Nejati A (2024). PPARgamma dependent PEX11beta counteracts the suppressive role of SIRT1 on neural differentiation of HESCs. PLoS One.

[399] Lu Y, Ding J, Huang J, Zhao Y (2023). ESE-1 mediates lipopolysaccharide-induced BV2 cell inflammation via upregulation of HMGB1 expression. J Biosci.

[400] Nerurkar PV, Johns LM, Buesa LM (2011). Momordica charantia (bitter melon) attenuates high-fat diet-associated oxidative stress and neuroinflammation. J Neuroinflammation.

[401] Wang Y, Chen Y, Yang J, Sun W, Zhang X (2025). Electro-acupuncture therapy alleviates post-stroke insomnia by regulating Sirt1 and the Nrf2-ARE pathway. Neuromolecular Med.

[402] Chen L, Xu W, Zhang Y, Chen H, Han Y (2023). Gandouling alleviates nerve injury through PI3K/Akt/FoxO1 and Sirt1/FoxO1 signaling pathway to inhibit autophagy in the rats model of Wilson’s disease. Brain Behav.

[403] Hamzeh O, Halaji M, Ghasemi-Kasman M, Parsian H, Rostami-Mansoor S (2025). Comparative effects of co-administration of Lactobacillus casei and Bifidobacterium breve vs. monotherapy on hippocampal neurodegeneration in a D-galactose-induced aging model. Mol Neurobiol.

[404] Khan M, Ullah R, Rehman SU (2019). 17β-Estradiol modulates SIRT1 and halts oxidative stress-mediated cognitive impairment in a male aging mouse model. Cells.

[405] Lu Y, Tan L, Wang X (2019). Circular HDAC9/microRNA-138/sirtuin-1 pathway mediates synaptic and amyloid precursor protein processing deficits in Alzheimer’s disease. Neurosci Bull.

[406] Maiese K (2015). FoxO proteins in the nervous system. Anal Cell Pathol (Amst).

[407] Sharma N, Shandilya A, Kumar N, Mehan S (2021). Dysregulation of SIRT-1 signaling in multiple sclerosis and neuroimmune disorders: a systematic review of SIRTUIN activators as potential immunomodulators and their influences on other dysfunctions. Endocr Metab Immune Disord Drug Targets.

[408] Balan V, Miller GS, Kaplun L (2008). Life span extension and neuronal cell protection by Drosophila nicotinamidase. J Biol Chem.

[409] Beegum F, P VA, George KT (2022). Sirtuins as therapeutic targets for improving delayed wound healing in diabetes. J Drug Target.

[410] Cao Y, Kanta JM, Bishop CA, Kiens B, Fritzen AM, Kleinert M (2025). Dietary medium-chain triacylglycerols in metabolic regulation. Trends Endocrinol Metab.

[411] El-Beltagy A, Saleh AMB, Attaallah A, Gahnem RA (2021). Therapeutic role of Azadirachta indica leaves ethanolic extract against diabetic nephropathy in rats neonatally induced by streptozotocin. Ultrastruct Pathol.

[412] Fang X, Song J, Chen Y (2023). LncRNA SNHG1 knockdown inhibits hyperglycemia induced ferroptosis via miR-16-5p/ACSL4 axis to alleviate diabetic nephropathy. J Diabetes Investig.

[413] Scrimieri R, Locatelli L, Cazzaniga A (2023). Ultrastructural features mirror metabolic derangement in human endothelial cells exposed to high glucose. Sci Rep.

[414] Wang L, Li L, Liu J (2025). Associated factors and principal pathophysiological mechanisms of type 2 diabetes mellitus. Front Endocrinol (Lausanne).

[415] Baser KHC, Haskologlu IC, Erdag E (2025). Molecular links between circadian rhythm disruption, melatonin, and neurodegenerative diseases: an updated review. Molecules.

[416] Fernandes J, Uppal K, Liu KH (2023). Antagonistic interactions in mitochondria ROS signaling responses to manganese. Antioxidants (Basel).

[417] Lee K, Lee D, Jeong H, Kim JY, Shim JJ, Lee JH (2025). Lactiplantibacillus plantarum HY7715 alleviates restraint stress-induced anxiety-like behaviors by modulating oxidative stress, apoptosis, and mitochondrial function. Int J Mol Sci.

[418] Lei X, Wen D, Huang Z (2025). Icariin attenuates oxidative stress via SIRT1/PGC-1α pathway in SAH mice. Exp Neurol.

[419] Li Y, Yan X, Xu R (2025). Ginsenoside Rf Suppresses oxidative stress–induced insomnia by modulating mitophagy through the AMPK/mTOR signaling pathway. J Food Biochem.

[420] Qi K, Mu Y, Hu Y, Li J, Liu J (2025). Comprehensive landscape of cell death mechanisms: from molecular cross-talk to therapeutic innovation in oncology. Front Cell Dev Biol.

[421] Gupta S, Afzal M, Agrawal N (2025). Harnessing the FOXO-SIRT1 axis: insights into cellular stress, metabolism, and aging. Biogerontology.

[422] Ou X, Lee MR, Huang X, Messina-Graham S, Broxmeyer HE (2014). SIRT1 positively regulates autophagy and mitochondria function in embryonic stem cells under oxidative stress. Stem Cells.

[423] Ungvari Z, Labinskyy N, Mukhopadhyay P (2009). Resveratrol attenuates mitochondrial oxidative stress in coronary arterial endothelial cells. Am J Physiol Heart Circ Physiol.

[424] Lin CL, Huang WN, Li HH (2015). Hydrogen-rich water attenuates amyloid β-induced cytotoxicity through upregulation of Sirt1-FoxO3a by stimulation of AMP-activated protein kinase in SK-N-MC cells. Chem Biol Interact.

[425] Sun Q, Jia N, Wang W, Jin H, Xu J, Hu H (2014). Activation of SIRT1 by curcumin blocks the neurotoxicity of amyloid-β25-35 in rat cortical neurons. Biochem Biophys Res Commun.

[426] Hou J, Chong ZZ, Shang YC, Maiese K (2010). Early apoptotic vascular signaling is determined by Sirt1 through nuclear shuttling, forkhead trafficking, bad, and mitochondrial caspase activation. Curr Neurovasc Res.

[427] Gao K, Chen C, Ke X (2023). Improvements of age-related cognitive decline in mice by Lactobacillus helveticus WHH1889, a novel strain with psychobiotic properties. Nutrients.

[428] Maiese K (2019). Knowing when to trust your gut: the intimate relationship between neurodegenerative diseases and the brain-gut axis. Curr Neurovasc Res.

[429] Mikó E, Kovács T, Sebő É (2019). Microbiome-microbial metabolome-cancer cell interactions in breast cancer-familiar, but unexplored. Cells.

[430] Martins B, Vieira M, Delerue-Matos C, Grosso C, Soares C (2022). Biological potential, gastrointestinal digestion, absorption, and bioavailability of algae-derived compounds with neuroprotective activity: a comprehensive review. Mar Drugs.

[431] Su H, Wang WJ, Zheng GD (2022). The anti-obesity and gut microbiota modulating effects of taxifolin in C57BL/6J mice fed with a high-fat diet. J Sci Food Agric.

[432] Zeng Y, Wu Q, Guo M (2025). Gut microbiota-derived imidazole propionate: an emerging target for the prevention and treatment of cardiometabolic diseases. Front Endocrinol (Lausanne).

[433] Vikram A, Kim YR, Kumar S (2016). Vascular microRNA-204 is remotely governed by the microbiome and impairs endothelium-dependent vasorelaxation by downregulating Sirtuin1. Nat Commun.

[434] Caron AZ, He X, Mottawea W (2014). The SIRT1 deacetylase protects mice against the symptoms of metabolic syndrome. FASEB J.

[435] Lei Q, Wu T, Wu J (2021). Roles of α-synuclein in gastrointestinal microbiome dysbiosis-related Parkinson’s disease progression (Review). Mol Med Rep.

[436] Odorskaya MV, Mavletova DA, Nesterov AA (2024). The use of omics technologies in creating LBP and postbiotics based on the Limosilactobacillus fermentum U-21. Front Microbiol.

[437] Wang H, Yang F, Zhang S, Xin R, Sun Y (2021). Genetic and environmental factors in Alzheimer’s and Parkinson’s diseases and promising therapeutic intervention via fecal microbiota transplantation. NPJ Parkinsons Dis.

[438] Najjar RS, Turner CG, Wong BJ, Feresin RG (2021). Berry-derived polyphenols in cardiovascular pathologies: mechanisms of disease and the role of diet and sex. Nutrients.

[439] Zeng Y, Wu Y, Zhang Q, Xiao X (2024). Crosstalk between glucagon-like peptide 1 and gut microbiota in metabolic diseases. mBio.

